# Glucocorticoid-Induced alterations in DNA methylation in the *H19* promoter of Bone Marrow-Derived Mesenchymal Stem Cells are associated with the pathogenesis of osteonecrosis

**DOI:** 10.1371/journal.pone.0345372

**Published:** 2026-03-27

**Authors:** Song Gong, Lizhi Han, Yang Wang, Yong Feng, Ruoyu Wang, Shizhan Zhang, Kun Nie, Bo Wang, Fei Du, Jianzhong Guan, Lu Zhang, Jiangtao Liu, Weihua Xu

**Affiliations:** 1 Department of Orthopaedics, The Central Hospital of Wuhan, Tongji Medical College, Huazhong University of Science and Technology, Wuhan, China; 2 Department of Orthopaedics, Union Hospital, Tongji Medical College, Huazhong University of Science and Technology, Wuhan, China; 3 Department of Orthopedics, The First Affiliated Hospital of Bengbu Medical University, Anhui Key Laboratory of Tissue Transformation, Bengbu Medical University, Bengbu, Anhui Province, P.R. China; 4 Department of Rehabilitation, Wuhan No.1 Hospital, Tongji Medical College, Huazhong University of Science and Technology, Wuhan, Hubei Province, China; 5 Institute of Hematology, Union Hospital, Tongji Medical College, Huazhong University of Science and Technology, Wuhan, China; Fujita Health University: Fujita Ika Daigaku, JAPAN

## Abstract

**Background:**

Glucocorticoid (GC)-induced osteonecrosis of the femoral head (ONFH) involves bone marrow-derived mesenchymal stem cell (BMSC) apoptosis and dysregulated osteo-adipogenic differentiation. While aberrant *H19* promoter methylation and expression have been linked to various bone metabolic disorders such as osteoporosis and osteosarcoma, their specific role in the pathogenesis of GC-induced ONFH remains largely unexplored.

**Methods:**

We analyzed *H19* promoter methylation, *DNMTs*, and *H19* expression in human ONFH BMSCs. Roles of *Dnmt1* and *H19* in osteogenic/adipogenic differentiation were assessed using staining (Alizarin Red/Oil Red O) and pathway analysis. Effects of *Dnmt1* knockdown or *H19* overexpression were tested via BMSC implantation in a GC-induced ONFH rat model.

**Results:**

*H19* promoter hypomethylation caused *H19* overexpression in undifferentiated GC-ONFH BMSCs; expression decreased upon differentiation. *H19* and *Dnmt1* expression were negatively correlated. *Dnmt1* predominated among *DNMTs* in epigenetically regulating *H19* and reciprocally modulated differentiation (inhibiting osteogenesis, promoting adipogenesis). Conversely, *H19* promoted osteogenesis and inhibited adipogenesis by suppressing *GSK-3β*, activating Wnt/β-catenin signaling. In the rat model, implanted BMSCs with *Dnmt1* knockdown or *H19* overexpression reduced empty lacunae, corrected the osteo-adipogenic imbalance, and delayed progression.

**Conclusion:**

The *Dnmt1*/*H19*/*GSK-3β* axis reciprocally regulates BMSC osteogenic and adipogenic differentiation in GC-induced ONFH, representing a novel epigenetic mechanism underlying GC-induced ONFH and a promising MSC-based therapeutic strategy for early-stage disease.

## Introduction

Osteonecrosis of the femoral head (ONFH) is a prevalent and refractory bone disorder that primarily affects adults under the age of 50 [1]. If left untreated, ONFH leads to osteocyte death and progressive collapse of the articular surface, culminating in degenerative arthritis characterized by severe hip pain and dysfunction [2,3]. Long-term and/or excessive glucocorticoid (GC) use is the most common cause of non-traumatic ONFH [4,5], accounting for approximately 30−40% of all ONFH cases. The prevalence of GC-induced ONFH among patients receiving high-dose, long-term steroid therapy is estimated to range from 5% to 40%, highlighting a significant patient population at risk [6,7].Since the onset of the global COVID-19 pandemic, GCs have been widely utilized as anti-inflammatory adjuvants to mitigate hyperinflammation. Consequently, the incidence of GC-induced ONFH is expected to rise further, as suggested by numerous case series reporting an increased occurrence of ONFH following high-dose glucocorticoid therapy for severe COVID-19 [8,9]. Although large-scale population-based studies are needed to determine the exact incidence, this emerging clinical trend warrants heightened awareness and monitoring. Although the precise pathogenesis of GC-induced ONFH remains elusive, it is believed to result from the interplay of multiple factors, including genetic and epigenetic alterations, cell apoptosis and autophagy, endothelial dysfunction, and coagulopathy [10]. Among these, apoptosis and dysregulated osteo-adipogenic differentiation of bone marrow-derived mesenchymal stem cells (BMSCs) due to pharmacological GC exposure are considered key contributors to ONFH onset and progression [10,11].

Mesenchymal stem cells (MSCs) are non-hematopoietic stem cells with self-renewal capabilities, multilineage differentiation potential (including osteogenic, adipogenic, and chondrogenic lineages), and immunomodulatory properties [12,13]. Furthermore, MSCs exhibit several advantages, such as low immunogenicity, minimal risk of infection and tumorigenesis, and ease of isolation and expansion [14]. These attributes render MSCs ideal candidates for regenerative medicine and tissue engineering applications [15]. While endogenous GCs are essential for skeletal development, bone anabolism, and bone mass maintenance, prolonged exposure to pharmacological GC doses suppresses osteoblast differentiation and bone formation, leading to skeletal complications such as osteonecrosis, osteoporosis, and fractures [11,16]. Due to chronic GC exposure, BMSCs isolated from patients with GC-induced ONFH exhibit intrinsic osteogenic defects and exhibit a preferential differentiation toward adipogenic or chondrogenic lineages [17].

Long non-coding RNAs (lncRNAs) constitute a class of heterogeneous, non-protein-coding transcripts exceeding 200 nucleotides in length and are pervasively transcribed from mammalian genomes [18,19]. LncRNAs have emerged as crucial epigenetic regulators that modulate gene expression and influence diverse biological processes, including cell proliferation, differentiation, and apoptosis [20]. LncRNA *H19* (*H19*), transcribed from the *H19/IGF2* gene locus, is a highly conserved imprinted gene imprinted gene, normally expressed monoallelically from the maternal allele, enriched in CpG dinucleotides (referred to as CpG islands) in its promoter region [21]. This imprinting is controlled by the Imprinting Control Region (ICR), whose methylation on the paternal allele silences *H19* and allows expression of the adjacent insulin-like growth factor 2 (*IGF2*) gene. In recent years, *H19* has gained considerable attention as a key regulator of various cellular processes, including the osteogenic and adipogenic differentiation of MSCs [22,23]. Studies have indicated that dysregulated *H19* expression is closely associated with the pathogenesis of bone metabolism disorders such as osteosarcoma and osteoporosis [24,25]. For instance, in disuse osteoporosis, hypermethylation of the *H19* promoter leads to its downregulation and impaired osteogenesis [25]. Similarly, in calcific aortic valve disease, *H19* hypomethylation and overexpression promote osteogenic differentiation [26]. However, despite these established roles in other bone diseases, the epigenetic regulation of *H19*—particularly its methylation status and expression dynamics—in the context of GC-induced ONFH has not been elucidated. Given that GC exposure is a major risk factor for ONFH and profoundly alters BMSC differentiation, we hypothesized that GC-induced epigenetic changes in *H19* may contribute to the imbalance between osteogenesis and adipogenesis characteristic of ONFH.

DNA methylation, which involves the addition of a methyl group (CH_3_) to the carbon-5 position of the cytosine ring, is catalyzed by DNA methyltransferases (*DNMTs*) and represents one of the most critical and stable epigenetic modifications governing gene expression [27]. Typically, CpG islands are regions of the genome where DNA methylation is generally low, whereas methylation levels are much higher in the remainder of the genome, such as in repetitive elements and gene deserts. However, when methylation does occur at CpG islands in promoter regions, it is particularly significant as it leads to transcriptional repression and gene downregulation. This context-dependent methylation pattern underscores the critical role of promoter CpG island methylation in epigenetic regulation of gene expression. In mammals, three catalytically active *DNMTs*—*Dnmt1*, *Dnmt3a*, and *Dnmt3b*—regulate DNA methylation. *Dnmt1* is primarily responsible for maintaining DNA methylation patterns following replication, whereas *Dnmt3a* and *Dnmt3b* regulate *de novo* DNA methylation during development [28]. DNA methylation has been reported to play a pivotal role in the osteogenic and adipogenic differentiation of MSCs [29,30].

The Wnt/β-catenin signaling pathway is fundamental to MSC lineage commitment and bone metabolism [31]. Activation of this pathway occurs when Wnt ligands bind to cell surface receptors, leading to the suppression of glycogen synthase kinase-3β (*GSK-3β*) activity, subsequent hypophosphorylation of β-catenin, and inhibition of β-catenin degradation. This results in β-catenin accumulation in the cytoplasm and its subsequent translocation into the nucleus, where it binds to T-cell factor/lymphoid enhancer factor (TCF/LEF) complexes and promotes the transcription of Wnt target genes [32,33]. Previous studies have demonstrated that GC exposure inhibits *GSK-3β* phosphorylation at Ser9, thereby activating *GSK-3β* in both diabetic and non-diabetic models [34,35]. Furthermore, GC-induced inactivation of the Wnt/β-catenin signaling pathway plays a crucial role in impaired osteogenesis and enhanced adipogenesis of MSCs [11,16,36–38].

In this study, we investigated whether aberrant methylation of the *H19* promoter was present in BMSCs of patients with GC-induced ONFH and assessed the effects of alterations in DNMT activity and *H19* expression on osteogenic and adipogenic differentiation in these cells. Furthermore, we examined whether the Wnt/β-catenin signaling pathway was involved in *H19*-mediated regulation of osteogenesis and adipogenesis. We sought to validate whether abnormal methylation of the *H19* promoter and aberrant *H19* expression in BMSCs, induced by GC administration, were closely associated with the pathogenesis of GC-induced ONFH. To translate our in vitro findings in human BMSCs to an in vivo setting, we established a rat model of GC-induced ONFH. Subsequently, the potential preventative effects of intra-femoral injection of rat BMSCs from early-stage ONFH, which had been genetically modified to prevent aberrant DNA methylation or restore *H19* expression, were evaluated in GC-induced ONFH rats. This approach demonstrated the feasibility of generating autologous genetically modified BMSCs, providing a convenient and reliable source for stem cell therapy in early-stage ONFH.

## Materials and methods

### Patient recruitment and grouping

The diagnosis of ONFH was confirmed through preoperative radiography and magnetic resonance imaging (MRI) in accordance with the Association Research Circulation Osseous (ARCO) staging system [39], while femoral neck fractures (FNF) were confirmed by X-ray imaging. The inclusion criteria for patients diagnosed with GC-induced ONFH were as follows: ARCO stage III or IV ONFH, age between 20 and 60 years, and requirement of total hip arthroplasty (THA) for treatment. The GC exposure threshold was defined as at least 1800 mg of prednisolone or its equivalent administered over a minimum duration of four weeks, with no history of alcohol consumption or smoking. The inclusion criteria for patients in the control (FNF) group were as follows: age between 20 and 60 years, and requirement of THA for treatment of the fracture. In addition, patients with FNF had no history of GC usage. The exclusion criteria for both groups included immunodeficiency, active infection or infectious disease, poorly controlled chronic conditions (e.g., hypertension, diabetes, heart disease, cancer), and hematological disorders. Eligible patients were recruited between August 3st, 2021 and March 31st, 2022 at the authors’ institution. Ten patients with GC-induced ONFH were assigned to the ONFH group, while ten individuals with FNF served as the control group. A detailed comparison between patients in both groups is presented in Table 1, and the demographic and clinical characteristics of each patient are summarized in Additional file 1: S1 Table.

**Table 1:** Comparison between patients in the ONFH group and the control group.

Demographics	control group (n = 10)	ONFH group (n = 10)	P value
Males	5	5	1.0
Females	5	5	
Age (years)	52.0 ± 2.0	52.0 ± 1.9	> 0.9999
BMI (kg/m^2^)	22.37 ± 0.50	24.01 ± 0.51	0.0343
B.G. (mmol/L)	5.07 ± 0.26	4.82 ± 0.13	0.3984

B.G., Blood Glucose

### Isolation and culture of human BMSCs

Primary human BMSCs (hBMSCs) were isolated from bone marrow aspirates obtained during THA and cultured using plastic adherence, as described in our previous study [40]. Briefly, cells were isolated from bone marrow using density gradient centrifugation, resuspended in DMEM-F12 complete culture medium (Gibco, USA) supplemented with 10% fetal bovine serum (FBS; Gibco, Australia) and 1% penicillin-streptomycin (Solarbio, China), and incubated in a humidified atmosphere at 37°C with 5% CO2. After 2–3 days, non-adherent cells were removed by washing with phosphate-buffered saline (PBS; Boster, China), while adherent cells were cultured for 10–14 days until they reached 80%–90% confluence. The cells were then trypsinized with 0.25% trypsin and 0.02% EDTA (Gibco, USA) and passaged at an appropriate dilution ratio (1:2 or 1:3) for initial subculture. Cells were subsequently expanded through successive passages in vitro, and second- or third-passage cells were used for further experiments to minimize the effects of cellular senescence and passage-induced epigenetic drift on experimental outcomes.

### Treatment with 5-aza-dC or Tideglusib and cell viability assay

The DNA demethylating agent 5-aza-2’-deoxycytidine (5-aza-dC; A3656) and the *GSK-3β* inhibitor tideglusib (SML0339) were obtained from Sigma-Aldrich, dissolved in dimethyl sulfoxide (DMSO; Solarbio), and further diluted in PBS. For the CCK-8 assay, hBMSCs were seeded into a 96-well plate (1 × 10^4^ cells/well), allowed to adhere for 24 h, and subsequently treated with varying concentrations of 5-aza-dC or tideglusib for 24, 48, and 72 h. The culture medium was then replaced with 100 μl of fresh serum-free medium (DMEM-F12 without FBS) containing 10% Cell Counting Kit-8 (CCK-8; Dojindo, Japan) and incubated at 37°C for 2 h. Optical density (OD) at 450 nm was measured using a microplate reader (Thermo Scientific, CA, USA) to assess cell proliferation. For osteogenic or adipogenic differentiation, cells were seeded into six-well plates (2 × 10^4^ cells/cm^2^) and treated with a specific concentration of 5-aza-dC or tideglusib for 72 h. The culture medium was subsequently replaced with osteogenic or adipogenic differentiation medium.

### Osteogenic and Adipogenic differentiation protocol for BMSCs

To induce osteogenic or adipogenic differentiation *in vitro*, hBMSCs were seeded at a density of 2 × 10^4^ cells/cm^2^ in six-well plates and cultured in DMEM-F12 medium supplemented with 10% FBS. Once the cells reached 60%–70% confluence, the medium was replaced with osteogenic differentiation medium (Cyagen, Guangzhou, China), supplemented with 10% FBS, 50 μg/mL L-ascorbic acid, 10 mM β-glycerophosphate, 100 nM dexamethasone, and 1% penicillin-streptomycin. The osteogenic induction medium was refreshed every 2–3 days, and cells were harvested for mRNA expression analysis at days 3, 7, and 14, for protein expression analysis at day 14, and for Alizarin Red S staining at day 14 post-induction. For adipogenic differentiation, once cells reached 100% confluence, the culture medium was replaced with adipogenic induction medium (Cyagen, Guangzhou, China) according to the manufacturer’s protocol. Adipogenic induction media A and B contained standard medium supplemented with 10% FBS, 0.2% insulin, 1% L-glutamine, and 1% penicillin-streptomycin, while medium A also included 0.1% IBMX, 0.1% dexamethasone, and 0.1% rosiglitazone. Cells were initially cultured in adipogenic induction medium A for 3 days, followed by medium B for 24 h, with this cycle repeated for 3 cycles (a total of 12 days). Cells were then harvested for mRNA expression analysis at days 3, 7, and 12, for protein expression analysis at day 12, and for Oil Red O staining at day 21 post-induction.The difference in confluence requirements for inducing differentiation is based on established protocols optimized for each lineage. For osteogenic differentiation, induction at sub-confluence (60–70%) is standard as it promotes cell proliferation and matrix deposition essential for bone nodule formation. In contrast, adipogenic differentiation is typically induced at 100% confluence (post-confluence), as growth arrest and cell-to-cell contact are critical initiating signals for adipocyte commitment and lipid droplet accumulation [41,42].

### Alizarin red staining and quantitative analysis

Extracellular matrix mineralization and calcium deposition were assessed using Alizarin Red S (ARS) staining following induction of osteogenic differentiation of hBMSCs for 14 days. Cells were fixed with 4% paraformaldehyde (PFA) for 20 min, stained with 1% ARS solution (pH = 4.2; Solarbio) for 5 min at room temperature, and rinsed three times with deionized distilled water. Staining was observed and documented under an inverted optical microscope (Olympus, Japan). For quantification of matrix mineralization, stained cells were destained and solubilized using 10% cetylpyridinium chloride (Sigma-Aldrich, USA) for 1 h at room temperature, and absorbance at OD 570 nm was measured spectrophotometrically using a microplate reader.

### Oil Red O staining and quantification

Following induction of adipogenic differentiation of hBMSCs for three weeks, Oil Red O (ORO) staining was performed to assess intracellular lipid accumulation in mature adipocytes. The ORO working solution was prepared by diluting the ORO stock solution (Solarbio) with deionized distilled water at a 3:2 ratio and was filtered immediately prior to use. After thorough rinsing with PBS, cells were fixed with 4% PFA for 30 min and subsequently stained with the ORO working solution for 20 min at room temperature. Following staining, the cells were washed three times with distilled water to remove unbound dye, visualized under an inverted microscope, and photographed. For quantification of intracellular lipid content, 100% isopropanol (Hushi, Shanghai, China) was added to each stained well to extract the stained lipid, and the absorbance of each well was measured at 450 nm.

### RNA extraction, reverse transcription, and quantitative real-time polymerase chain reaction (qRT-PCR)

Total cellular RNA was extracted using the TRIzol reagent (Accurate Biotechnology Co., Ltd., Hunan, China) and reverse‐transcribed into cDNA using the *Evo M-MLV* RT Premix (Accurate Biotechnology) in accordance with the manufacturer’s protocol. Quantitative real-time PCR (qRT-PCR) was performed using the SYBR® Green Premix *Pro Taq* HS qPCR Kit (Accurate Biotechnology) on a QuantStudio7 real-time PCR system (Applied Biosystems, Carlsberg, USA). Relative lncRNA and mRNA expression levels were calculated using the 2^−ΔΔCt^ method, with β-actin serving as the endogenous control. All primer sequences used for qRT-PCR are listed in Additional file 2: S2 Table.

### Bisulfite sequencing

Total genomic DNA was extracted from hBMSCs using the Genomic DNA Kit (Tiangen Biotech Co. Ltd, China) in accordance with the manufacturer’s instructions. Bisulfite treatment of DNA, which converts unmethylated cytosine to uracil while preserving methylated cytosine residues, was conducted using the EZ DNA Methylation-Gold Kit (Zymo Research, USA) following the manufacturer’s guidelines. Bisulfite-modified DNA was purified using the QIAquick PCR purification kit (Qiagen, CA, USA). The promoter regions of *H19* were PCR-amplified from bisulfite-treated DNA and subjected to bisulfite DNA sequencing for quantification of CpG methylation. The following primers were used: forward primer, TAGGATTTTTGTGTTGTTGGAGATA; reverse primer, ACACCTATAAACAAATTCACCTCTC. This yielded a 196-bp product (−1599 to −1404), which contained 12 CG sites. The amplified sequences were cloned into the pMD19-T vector (Takara, Dalian, China), and multiple clones (typically 10 per sample) were picked for Sanger sequencing to account for methylation heterogeneity and potential allelic differences..The promoter methylation status of the *H19* gene was analyzed using the Quantitative Tool for Methylation Analysis (QUMA) software [43], which calculates the average methylation percentage across all sequenced clones for a given sample.

### Western blot analysis

Cells were washed three times with cold PBS and lysed with radioimmunoprecipitation assay (RIPA) buffer (Solarbio, China) containing protease/phosphatase inhibitor cocktails (Beyotime, China) on ice for 30 min. The supernatants were collected, and total protein concentration was determined using a BCA protein assay kit (Elabscience, China). Equal amounts of protein were separated on a 10% sodium dodecyl sulfate-polyacrylamide gel electrophoresis (SDS-PAGE) and transferred onto a polyvinylidene fluoride (PVDF) membrane (Millipore, USA). After blocking with 5% defatted milk in Tris-buffered saline with Tween 20 (TBST) at room temperature for 2 h, membranes were incubated overnight at 4°C with specific primary antibodies. The primary antibodies used in this study included anti-RUNX2 (1:500, bs-1134R, Bioss-bio, China), anti-COL1A1 (1:1000, bsm-52478R, Bioss-bio, China), anti-FABP4 (1:1000, ab93945, Abcam), anti-PPARγ (1:500, ARG55241, Arigobio), anti-*Dnmt1* (1:1000, ab188453, Abcam), anti-*Dnmt3a* (1:1500, ab228691, Abcam), anti-*Dnmt3b* (1:500, ab119282, Abcam), anti-phospho-*GSK-3β* (Ser9) (1:10000, ab75814, Abcam), anti-total *GSK-3β* (1:1000, ab93926, Abcam), anti-active (non-phosphorylated) β-catenin (1:1000, ab246504, Abcam), and anti-total β-catenin (1:5000, ab32572, Abcam). After washing with TBST, membranes were incubated with the corresponding horseradish peroxidase (HRP)-labeled secondary antibodies (Elabscience) at room temperature for 1 h. β-Actin (1:10000, ab8226, Abcam) was used as a protein loading control. Protein expression levels were analyzed using ImageJ software (NIH, USA).

### Immunofluorescence

For cellular immunofluorescence (IF) experiments, hBMSCs were seeded on 0.1% gelatin-coated glass coverslips and cultured for 48 h. Cells were then washed with PBS, fixed in 4% PFA for 30 min, permeabilized in PBS containing 0.2% Triton X-100 (Solarbio) for 10 min, and blocked with 5% goat serum (AR0009, Boster, China) for 30 min at room temperature. Cells were incubated overnight at 4°C with primary antibodies against *Dnmt1* (1:1000, ab188453, Abcam), followed by incubation with DyLight 594-conjugated goat anti-rabbit IgG (1:200, A23420, Abbkine) at room temperature for 2 h. Finally, nuclei were stained with DAPI (10 μg/mL; C0065, Solarbio), and slides were mounted using an antifade mounting medium (S2100, Solarbio).

### Transfections

Small interfering RNAs (siRNAs) targeting *H19*, *Dnmt1*, *Dnmt3a*, and *Dnmt3b* were transfected into hBMSCs from the ONFH group according to the manufacturer’s protocol (Riobio, Guangzhou, China). Non-targeting control siRNAs (Riobio) served as negative controls. Briefly, cells were transfected with either 50 nM siRNAs or control siRNA for 48 h using the RiboFECTTM CP reagent (Riobio). For overexpression, hBMSCs were transfected with *H19* or *Dnmt1* expression plasmids (pENTER vector; Vigene Biosciences, China) using Lipofectamine 2000 reagent (Invitrogen, USA) for 48 h, with empty vector plasmids serving as negative controls (Invitrogen, USA). The siRNA sequences used for gene silencing are provided in Additional file 3: S3 Table.The siRNAs and shRNAs targeting H19, Dnmt1, Dnmt3a, and Dnmt3b were designed using validated algorithms from the supplier (Riobio, Guangzhou, China). The sequences were selected based on the following criteria: (1) specificity to the target gene mRNA sequence, (2) minimal off-target potential as assessed by BLAST analysis against the human or rat genome, and (3) prior validation of silencing efficiency in preliminary experiments. For each target gene, three independent siRNA/shRNA sequences were initially screened, and the most effective one (as confirmed by qRT-PCR or western blot) was used for subsequent experiments. The non-targeting control siRNA/shRNA was provided by the manufacturer and has been validated to not target any known gene in the human or rat transcriptome.

### Ethics approval and consent to participate

All human studies were approved by the Ethics Committee of Union Hospital, Tongji Medical College, Huazhong University of Science and Technology (Approval Number: UHCT-IEC-SOP-016-03-01). Written informed consent was obtained from all enrolled participants prior to the operative procedure. All animal procedures were conducted in accordance with a protocol approved by the Institutional Animal Care and Use Committee at Tongji Medical College, Huazhong University of Science and Technology (IACUC Number: 2706). All animal experiments were performed in strict compliance with the ARRIVE guidelines. I confirm that all methods were performed in accordance with the relevant guidelines. All procedures were performed in accordance with the ethical standards laid down in the 1964 Declaration of Helsinki and its later amendments.

### Establishment of the GC-Induced ONFH Rat Model

Ninety adult male Sprague Dawley (SD) rats (16–18 weeks old, weighing 400–500 g) were obtained from the Animal Experiment Center of Tongji Medical College, Huazhong University of Science and Technology. The GC-induced ONFH model was established using methylprednisolone (MPS) in combination with lipopolysaccharide (LPS), as previously described [44], with modifications. Briefly, rats received intraperitoneal (i.p.) injections of LPS (L2880, Sigma-Aldrich, USA) at 0.1 mg/kg once daily for three consecutive days. On days 4, 5, and 6, high-dose MPS (Pfizer Pharmaceutical, China) was administered intramuscularly (i.m.) at 100 mg/kg, followed by low-dose MPS (40 mg/kg, i.m.) three times per week for the subsequent three weeks. Throughout the experiment, all efforts were made to minimize suffering. Animals were monitored daily for signs of distress, such as reduced mobility, weight loss, or altered grooming behavior. Soft food and hydration gels were provided to support nutrition and hydration. Any animal showing severe distress was euthanized immediately to prevent unnecessary suffering.The model establishment process is illustrated in Figure 6A.

### Isolation, culture, and lentiviral transfection of Rat BMSCs

Primary BMSCs from Sprague Dawley rats (rBMSCs) were isolated from the bilateral femur and tibia that had undergone four weeks of MPS injections, as well as age-matched normal control rats, following a previously described method [45]. Briefly, the isolated cells were cultured in DMEM-F12 medium (Gibco) supplemented with 10% FBS (Gibco) and 1% penicillin–streptomycin (Solarbio) at 37°C in a humidified atmosphere containing 5% CO₂. Adherent cells were passaged upon reaching 80%–90% confluence. Cells from the first passage were harvested and utilized for subsequent experiments.

To achieve stable silencing of *Dnmt1* or overexpression of *H19* in BMSCs from rats that had completed four weeks of MPS injections, lentiviral transfection was performed. A short hairpin RNA (shRNA) targeting *Dnmt1* was synthesized and cloned into a pLVX vector (Cyagen, Guangzhou, China) for the knockdown of *Dnmt1* in rat BMSCs (rBMSCs). Additionally, a packaged *H19*-overexpressing lentiviral vector and its corresponding negative control were obtained from Cyagen Biosciences. All lentiviral vectors were transfected into rBMSCs at a multiplicity of infection (MOI) of 50. Cells from the first passage were plated in six-well plates at a density of 2 × 10⁴ cells/cm². Upon reaching 40%–50% confluence, cells were transfected in fresh serum-free medium containing 5 μg/ml polybrene (Cyagen) to enhance transfection efficiency, in accordance with the manufacturer’s instructions. The culture medium was replaced with DMEM-F12 containing 10% FBS six hours post-transfection. The knockdown efficiency of *Dnmt1* and the overexpression efficiency of *H19* were confirmed by western blot and qRT-PCR, respectively. The shRNA sequences used for *Dnmt1* gene silencing are provided in Additional file 4: S4 Table.

### Animal grouping, cell labeling, and BMSC transplantation

To investigate the effects of *Dnmt1* silencing and *H19* overexpression on the progression of GC-induced ONFH, rBMSCs transfected with either shRNA-*Dnmt1* lentivirus or *H19*-overexpressing lentiviral vector, along with their respective negative controls (Lenti-Ctrl), were transplanted into MPS-treated rats via intra-femoral injection. MPS-treated and normal rats that received sham intra-femoral injections of vehicle (PBS) were used as model and normal controls, respectively. MPS-treated rats that received intra-femoral injections of BMSCs from normal rats served as positive controls, as previously demonstrated to be effective [46]. Consequently, rats were randomly assigned to the following groups for *in vivo* studies (n = 6 per group): (a) normal control (NC) + vehicle-treated group, (b) MPS + vehicle-treated group, (c) MPS + Lenti-Ctrl (sh-Ctrl or oe-Ctrl) rBMSCs-treated group, (d) MPS + shRNA-*Dnmt1* (sh-*Dnmt1*) or *H19*-overexpression (oe-*H19*) rBMSCs-treated group, and (e) MPS + NC rBMSCs-treated group.Cell labeling and rBMSC transplantation were conducted as described previously [46,47]. Briefly, when rBMSCs reached 50%–60% confluence, 5-bromo-2-deoxyuridine (BrdU; Sigma) was added to the culture medium at a final concentration of 10 μM for 72 hours to label proliferating cells. The cells were then harvested upon reaching approximately 90% confluence. For rBMSC transplantation, rats were anesthetized intraperitoneally with 3% sodium pentobarbital (1 ml/kg), and the depth of anesthesia was confirmed by absence of pedal reflex. The surgical site was disinfected. A longitudinal incision was made over the patella to expose the femoral condyle through patella dislocation. A puncture needle was inserted into the femoral intercondylar region, penetrating the bone marrow cavity until reaching the proximal femur. Labeled rBMSCs were washed three times with PBS, resuspended in 200 μl PBS, and injected intra-femorally into each rat (2 × 10⁵ cells per 10 g body weight) in the designated treatment group at the fourth week of MPS injections. Following the injection, the puncture needle was maintained in place for one minute before being slowly withdrawn, and the puncture site was sealed with sterilized bone wax. Post-operative analgesia was provided using buprenorphine (0.05 mg/kg) subcutaneously every 12 hours for 48 hours. Rats in the NC and MPS groups received an intra-femoral injection of an equivalent volume of PBS. At the end of the experiment, all rats were euthanized by overdose of sodium pentobarbital (150 mg/kg, i.p.) followed by cervical dislocation to ensure death. The rats were euthanized, and femoral head samples were collected at the sixth week following BMSC transplantation (as illustrated in Figure 6A).

### Micro-CT Scanning and analysis

Rat femoral head tissues were harvested six weeks after BMSC transplantation, fixed in 4% PFA, and scanned using a SkyScan 1176 Micro-CT scanner (Bruker MicroCT, Kontich, Belgium). The image resolution was set to 9 μm per pixel. Raw images were reconstructed and analyzed using the SkyScan software programs NRecon (version 1.7.4.6), DataViewer (version 1.5.6.2), and CT Analyser (version 1.18.8.0). Femoral head and neck slices were generated and reoriented in three planes (coronal, transverse, and sagittal) using the DataViewer software. The trabecular bone region of interest (ROI) was segmented from the bone marrow and analyzed to assess trabecular microstructure parameters, including bone volume per tissue volume (BV/TV), trabecular thickness (Tb.Th), trabecular number (Tb.N), and trabecular separation (Tb.Sp), using CT Analyser software.

### Hematoxylin and Eosin staining and immunohistochemistry

At week six post-BMSC transplantation, rat femoral head samples were collected, fixed in 4% PFA for three days, decalcified in 0.5 M ethylenediaminetetraacetic acid (EDTA) solution (G1105, Servicebio, China) at pH 8.0 for four weeks, dehydrated, and embedded in paraffin. The paraffin-embedded tissue sections were cut into serial coronal slices with a thickness of approximately 4 μm. Some sections were subjected to hematoxylin and eosin (H&E; Servicebio) staining to evaluate trabecular structure, while others were processed for immunohistochemistry (IHC). For IHC staining, paraffin sections were deparaffinized, subjected to antigen retrieval, blocked, and incubated overnight at 4°C with primary antibodies against BrdU (1:100, 66241–1-Ig, Proteintech), COL1A1 (1:100, BA0325, Boster, China), and FABP4 (1:100, E-AB-60028, Elabscience). The sections were subsequently washed and incubated with appropriate secondary antibodies for one hour at room temperature. Immunohistochemical images were analyzed by quantifying the number of positive cells using ImageJ software.

### Statistical Analysis

Statistical analyses were performed using GraphPad Prism 8.0 (GraphPad Software, CA, USA). All quantitative data are presented as mean ± standard deviation (SD) based on a minimum of three independent experiments. For all experiments involving the comparison of more than two groups, one/two-way analysis of variance (ANOVA) was applied, followed by Bonferroni’s post-hoc test for multiple comparisons, to control the family-wise error rate and reduce the probability of Type I errors. Unpaired, two-tailed Student’s t-tests were reserved exclusively for planned comparisons between two groups only. The specific statistical test used for each experiment is detailed in the corresponding figure legend.A P-value of less than 0.05 was considered statistically significant, whereas P-values greater than 0.05 were regarded as statistically nonsignificant (ns) (*#P* > 0.05, *P < 0.05, **P < 0.01, ***P < 0.001, and ****P < 0.0001).

## Results

### Aberrant expression patterns of DNMTs and H19 and Aberrant Methylation of the H19 Promoter in hBMSCs of the ONFH Group

**Fig 1:**
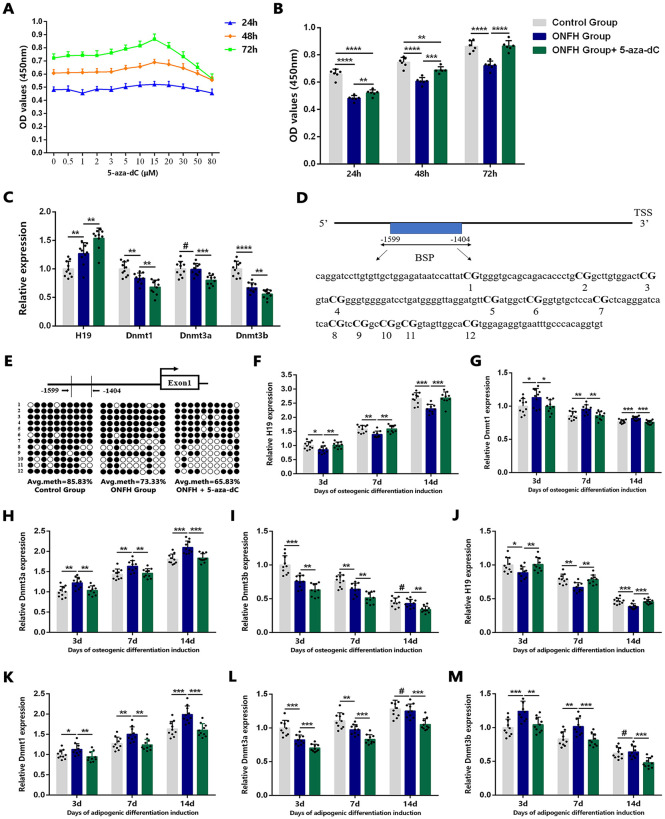
The DNMTs and H19 expression patterns and H19-promoter methylation status in hBMSCs of ONFH group. **(A)** When the concentration of 5-aza-dC reached 15 μM, cellular viability of hBMSCs from the ONFH group reached its peak at 24, 48, and 72 h (n = 6). **(B)** The cellular proliferative capacity was evaluated in hBMSCs of the control group, ONFH group, and 5-aza-dC treated group at 24, 48, and 72 h (n = 6). (C) qRT-PCR analysis revealed the relative expressions of *H19* and *DNMTs* in undifferentiated hBMSCs of the control group, ONFH group, and 5-aza-dC treated group (n = 10). **(D)** Schematic diagram showed the location of 12 CpG sites within the analyzed region of the *H19* promoter CpG island. **(E)** The BSP assay was used to analyze the methylation status of these CpG sites in the *H19* promoter in hBMSCs of the three group.In the resulting methylation graph, each column represents a single individual subject sample, and each row corresponds to one of the 12 specific CpG sites analyzed, ordered by their genomic location. Empty dots: unmethylated CpGs; black dots: methylated CpGs. (F–I) qRT-PCR analysis showed the relative expressions of *H19* **(F)**, *Dnmt1* **(G)**, *Dnmt3a* **(H)**, and *Dnmt3b* (I) at days 3, 7, and 14 during osteogenic differentiation (n = 10). (J–M) qRT-PCR analysis showed the relative expressions of *H19* **(J)**, *Dnmt1* **(K)**, *Dnmt3a* **(L)**, and *Dnmt3b* (M) at days 3, 7, and 14 during adipogenic differentiation (n = 10).Statistical analysis was performed using one/two-way ANOVA with Bonferroni’s post-hoc test. Data are presented as mean ± SD, #P > 0.05, *P < 0.05, **P < 0.01, ***P < 0.001, ****P < 0.0001.

A previous study [39] demonstrated that the isolated and cultured cells from both the ONFH and control groups conformed to the minimal criteria established by the International Society for Cell Therapy (ISCT) for MSCs [48], as verified by flow cytometry and trilineage differentiation (osteogenic, adipogenic, and chondrogenic differentiation) potentials. In this study, patients in the ONFH group received glucocorticoid (GC) treatment with a cumulative dose of at least 1800 mg prednisolone (or equivalent) over a minimum duration of four weeks, which is a well-established risk factor for osteonecrosis. To first establish a foundational link between DNA methylation and the observed phenotype in ONFH BMSCs, we utilized the DNA methyltransferase inhibitor 5-aza-2’-deoxycytidine (5-aza-dC). The goal of this experiment was twofold: first, to determine a non-cytotoxic concentration for subsequent long-term differentiation experiments, and second, to test the hypothesis that global DNA demethylation could reverse the aberrant gene expression and differentiation patterns characteristic of ONFH BMSCs. If GC-induced pathogenesis is mediated by DNA hypermethylation of specific promoters (e.g., *H19*), then pharmacological inhibition of *DNMTs* should alleviate this repression and restore normal function. To evaluate the effect of the DNMT inhibitor 5-aza-dC on cell viability in hBMSCs of the ONFH group, a CCK-8 assay was performed following 5-aza-dC treatment. Cellular viability peaked at a concentration of 15 μM and gradually declined with further increases in concentration at 24, 48, and 72 hours (Fig 1A). Additionally, the cellular viability of the ONFH group was significantly lower than that of the control group at each time point, suggesting that chronic exposure to high-dose glucocorticoids in vivo may impart lasting detrimental effects on the proliferative capacity and health of BMSCs, a characteristic intrinsic defect associated with the disease pathogenesis. However, this reduced viability was significantly enhanced following treatment with 15 μM 5-aza-dC (Fig 1B). Consequently, 15 μM was selected as the optimal concentration for subsequent 5-aza-dC treatment experiments.

To determine whether GC administration resulted in aberrant expression patterns of *DNMTs* and *H19*, along with abnormal methylation of the *H19* promoter in hBMSCs, qRT-PCR was utilized to assess the expression levels of *DNMTs* and *H19*, while bisulfite-sequencing PCR (BSP) was employed to analyze the DNA methylation status of the *H19* promoter. qRT-PCR results indicated that undifferentiated hBMSCs from the ONFH group exhibited lower expression levels of *Dnmt1* and *Dnmt3b* but higher expression levels of *H19* compared to the control group. No significant difference was observed in *Dnmt3a* expression between the two groups (Fig 1C). While a negative correlation with *H19* expression was observed for both DNMT1 and DNMT3B, subsequent functional experiments were designed to identify the primary regulator. Moreover, treatment with 15 μM 5-aza-dC significantly reduced the expression levels of *Dnmt1*, *Dnmt3a*, and *Dnmt3b* while markedly increasing *H19* expression in undifferentiated hBMSCs of the ONFH group (Fig 1C). BSP analysis further revealed hypomethylation of the *H19* promoter, corresponding to its increased expression, in undifferentiated hBMSCs of the ONFH group, with methylation levels further decreased following 5-aza-dC treatment (Fig 1D, 1E).

Next, the expression profiles of *H19* and *DNMTs* in differentiated hBMSCs were examined using qRT-PCR. The results demonstrated that *H19* expression progressively increased in hBMSCs from both groups at days 3, 7, and 14 during osteogenic differentiation (Fig 1F), whereas it gradually declined at these time points during adipogenic differentiation (Fig 1J). Notably, in contrast to the elevated *H19* expression observed in undifferentiated hBMSCs of the ONFH group, its expression levels were lower in cells that underwent osteogenic or adipogenic differentiation at each time point, while 5-aza-dC treatment effectively restored the decreased *H19* expression (Fig 1F, 1J). Among the three *DNMTs*, *Dnmt3a* and *Dnmt3b* exhibited substantial variability in expression levels in differentiated hBMSCs between the two groups (Fig 1H, 1I, 1L, 1M). However, *Dnmt1* expression remained consistently higher in differentiated hBMSCs of the ONFH group compared to the control group throughout osteogenic and adipogenic induction (Fig 1G, 1K). These findings suggest a negative correlation between the expression of *H19* and *Dnmt1* in both undifferentiated and differentiated BMSCs between the control and ONFH groups.

### Dnmt1 knockdown induces hypomethylation of the H19 Promoter and Upregulates H19 Expression, Whereas Dnmt1 Overexpression Exerts Opposite Effects

**Fig 2:**
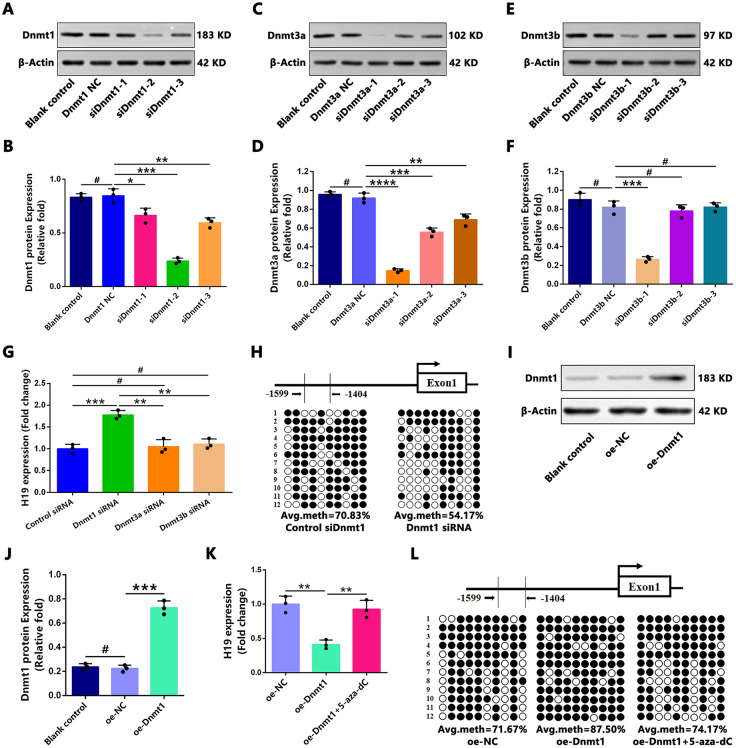
Dnmt1 was the major DNMT that epigenetically regulated H19 expression in ONFH group hBMSCs. **(A–F)** Representative western blot bands and relative quantification of proteins indicated that *Dnmt1* siRNA **(A–B)**, *Dnmt3a* siRNA **(C–D)**, and *Dnmt3b* siRNA (E–F) efficiently decreased the protein levels of *Dnmt1*, *Dnmt3a* and *Dnmt3b* in hBMSCs of the ONFH group. β-actin was used as a loading control. (G) qRT-PCR analysis showed the relative expression of *H19* in hBMSCs after DNMT knockdown. **(H)** BSP analysis was used to detect the methylation status of the *H19* promoter. **(I–J)** Representative western blot images and quantification of relative protein expression indicated that overexpression of *Dnmt1* by transfection efficiently increased *Dnmt1* expression in hBMSCs. β-actin was used as a loading control. (K) qRT-PCR analysis showed the expression differences of *H19* in hBMSCs from three different treatment groups. **(L)** BSP analysis was used to detect the methylation status of the *H19* promoter in hBMSCs of the three groups. Statistical analysis was performed using one/two-way ANOVA with Bonferroni’s post-hoc test. Data are presented as mean ± SD, n = 3 per group, #P > 0.05, *P < 0.05, **P < 0.01, ***P < 0.001, ****P < 0.0001. NC, negative control; si/siRNA: small interfering RNA; oe: overexpression.

Given that 5-aza-dC treatment resulted in hypomethylation of the *H19* promoter and elevated *H19* expression, we sought to identify the predominant DNMT responsible for epigenetic regulation of *H19* expression in ONFH group hBMSCs. Initially, the knockdown efficiencies of three independent DNMT-specific siRNAs were validated in hBMSCs of the ONFH group, with si*Dnmt1*−2, si*Dnmt3a*-1, and si*Dnmt3b*-1 identified as the most effective (Fig 2A-F). These siRNAs were subsequently selected for further experimentation. qRT-PCR analysis revealed that knockdown of *Dnmt1*, but not *Dnmt3a* or *Dnmt3b*, significantly upregulated *H19* expression (Fig 2G). Additionally, BSP analysis indicated that *Dnmt1* knockdown induced hypomethylation of the *H19* promoter (Fig 2H). Conversely, overexpression of *Dnmt1* in ONFH group hBMSCs was successfully achieved using a *Dnmt1* overexpression plasmid, as confirmed by western blot analysis (Fig 2I, 2J). Upregulation of *Dnmt1* expression resulted in decreased *H19* expression (Fig 2K) and hypermethylation of the *H19* promoter (Fig 2L). Notably, the hypermethylation of the *H19* promoter and reduced *H19* expression induced by *Dnmt1* overexpression were partially reversed by 5-aza-dC treatment (Fig 2K, 2L).

To investigate the subcellular localization of *Dnmt1* and *H19*, immunofluorescence (IF) staining of *Dnmt1* and qPCR analysis of *H19* in nuclear/cytoplasmic fractions were conducted. IF staining demonstrated that *Dnmt1* was predominantly localized in the nucleus of undifferentiated hBMSCs in both groups, with lower fluorescence intensity observed in ONFH group cells compared to control group cells (S1A Fig). Meanwhile, qPCR analysis of nuclear and cytoplasmic fractions revealed that *H19* was primarily distributed in the cytoplasm of undifferentiated hBMSCs in the control group, whereas it was predominantly localized in the nucleus in ONFH group cells (S1B Fig). The relative abundance of *H19* in each fraction was determined by calculating the ratio of its expression in the nucleus versus the cytoplasm (N/C ratio) for each sample. Expression levels in each fraction were normalized to the respective reference genes (U6 for nuclear RNA and GAPDH for cytoplasmic RNA) using the 2^(-ΔCt) method. The resulting N/C ratio for the ONFH group was significantly higher than that of the control group, indicating nuclear retention of *H19* in ONFH-derived cells.

These findings suggest that among the three *DNMTs*, *Dnmt1* plays a predominant role in the epigenetic regulation of *H19* expression in hBMSCs of patients with GC-induced ONFH. Furthermore, *Dnmt1* is primarily localized in the nucleus of undifferentiated hBMSCs, while GC administration may induce the translocation of *H19* from the cytoplasm to the nucleus in undifferentiated hBMSCs.

### Dnmt1 knockdown promotes osteogenesis and suppresses adipogenesis, While Dnmt1 Overexpression Exhibits Opposite Effects

**Fig 3:**
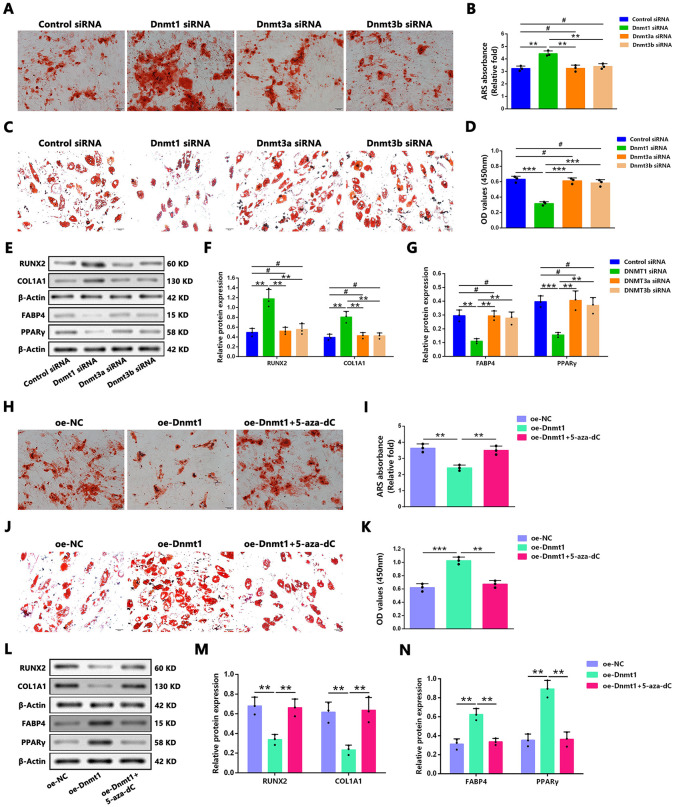
Dnmt1 negatively regulated osteoblast differentiation and positively regulated adipocyte differentiation in ONFH group hBMSCs. **(A–B)** ARS staining (A) and quantification (B) were performed to measure the calcium deposits in hBMSCs after 14 days of induction of osteogenic differentiation. Scale bars: 100 μm. **(C–D)** ORO staining (C) and quantification (D) were used to evaluate the intracellular lipid accumulation in hBMSCs after 21 days of induction of adipogenic differentiation. Scale bars: 50 μm. **(E–G)** After DNMT knockdown, the representative western blot band (E) and quantitative analysis showed the relative expressions of RUNX2 and COL1A1 **(F)**, as well as FABP4 and PPARγ **(G)**. β-actin was used as the loading control. (H–I) ARS staining (H) and quantification (I) were performed to measure the calcium deposits in hBMSCs. Scale bars: 100 μm. **(J-K)** ORO staining (J) and quantification (K) were used to evaluate the intracellular lipid accumulation in hBMSCs. Scale bars: 50 μm. **(L–N)** After overexpression of *Dnmt1*, the representative western blot band (L) and quantitative analysis showed the relative expressions of RUNX2 and COL1A1 (M) as well as FABP4 and PPARγ **(N)**. β-actin was used as the loading control. Statistical analysis was performed using one/two-way ANOVA with Bonferroni’s post-hoc test. Data are presented as mean ± SD, n = 3 per group, #P > 0.05, *P < 0.05, **P < 0.01, ***P < 0.001. NC, negative control; si/siRNA: small interfering RNA; oe: overexpression.

Given that the regulatory role of the three active *DNMTs* in mammals in the osteogenic and adipogenic differentiation of BMSCs in patients with GC-induced ONFH remains unclear, the effects of DNMT activity modulation on osteogenesis and adipogenesis were further investigated. ARS staining results indicated that *Dnmt1* knockdown, but not *Dnmt3a* or *Dnmt3b* knockdown, in hBMSCs of the ONFH group followed by induction of osteogenic differentiation significantly increased the number of mineralized nodules (Fig 3A and B) and markedly reduced the number of ORO-positive adipocytes following induction of adipogenic differentiation (Fig 3C and D). All uncropped scans of western blots are uploaded as supplementary files (Raw data). Furthermore, *Dnmt1* knockdown, but not *Dnmt3a* or *Dnmt3b* knockdown, in hBMSCs significantly increased the protein levels of the osteogenic markers RUNX2 and COL1A1 (Fig 3E and F) while markedly decreasing the protein levels of the adipogenic markers FABP4 and PPARγ (Fig 3E and G). Additionally, treatment of hBMSCs from the ONFH group with 5-aza-dC enhanced the mRNA expression of osteogenic markers and the formation of mineralized nodules while decreasing the mRNA expression of adipogenic markers and intracellular lipid accumulation (S2A-S2H Figs).

Conversely, *Dnmt1* overexpression reduced mineralized nodule formation (Figure 3H and I) and increased the number of ORO-positive adipocytes (Fig 3J and K). Consistent with these staining results, the protein levels of RUNX2 and COL1A1 were significantly reduced, whereas those of FABP4 and PPARγ were markedly increased following *Dnmt1* upregulation in hBMSCs (Fig 3L-N). Furthermore, the impaired osteogenic differentiation and enhanced adipogenic differentiation observed in hBMSCs with *Dnmt1* overexpression were mitigated by 5-aza-dC treatment, as demonstrated by ARS and ORO staining and western blot analysis (Fig 3H-N).

These findings collectively suggest that *Dnmt1* knockdown, but not *Dnmt3a* or *Dnmt3b* knockdown, in hBMSCs of the ONFH group promotes osteogenic differentiation while suppressing adipogenic differentiation, whereas *Dnmt1* overexpression inhibits osteogenic differentiation and enhances adipogenic differentiation.

### Silencing of H19 inhibits osteogenesis and enhances adipogenesis, while H19 Overexpression exhibits opposite effects

**Fig 4:**
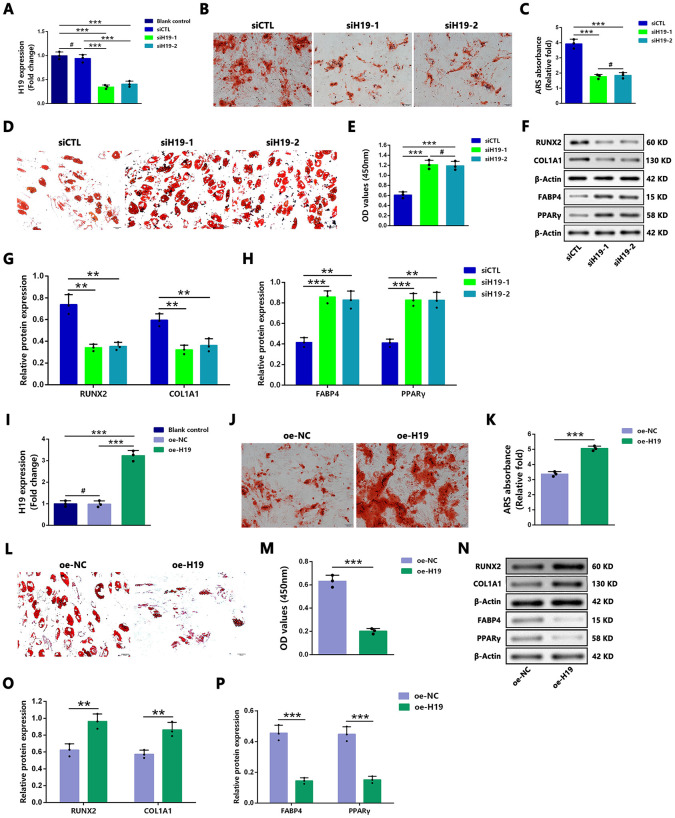
H19 positively regulated osteoblast differentiation and negatively regulated adipocyte differentiation in ONFH group hBMSCs. (A) qRT-PCR analysis verified the knockdown of *H19*. **(B–C)** ARS staining (B) and quantification (C) demonstrated the effect of *H19* silencing on the calcium deposits in hBMSCs. Scale bars: 100 μm. **(D–E)** ORO staining (D) and quantification (E) showed the effect of *H19* silencing on the intracellular lipid accumulation in hBMSCs. Scale bars: 50 μm. **(F–H)** Representative western blot image (F) and quantitative analysis showed the effect of *H19* silencing on the protein expression of RUNX2 and COL1A1 (G) as well as FABP4 and PPARγ **(H)**. β-actin was used as the loading control. (I) qRT-PCR analysis verified overexpression of *H19*. **(J–K)** ARS staining (J) and quantification (K) showed the effect of *H19* overexpression on the calcium deposits in hBMSCs. Scale bars: 100 μm. **(L–M)** ORO staining (L) and quantification (M) showed the effect of *H19* overexpression on the intracellular lipid accumulation in hBMSCs. Scale bars: 50 μm. **(N–P)** Representative western blot image (N) and quantitative analysis showed the effect of *H19* overexpression on the protein expressions of RUNX2 and COL1A1 (O) as well as FABP4 and PPARγ **(P)**. β-actin was used as the loading control. (A,C,E,G,H,I) Statistical analysis was performed using one/two-way ANOVA with Bonferroni’s post-hoc test. Data are presented as mean ± SD, n = 3 per group, #P > 0.05, **P < 0.01, ***P < 0.001. (K,M,O,P) Data are presented as mean ± SD, n = 3 per group, #P > 0.05, **P < 0.01, ***P < 0.001, unpaired Student’s t test. siCTL, scramble control siRNA; NC, negative control; oe: overexpression.

To elucidate the functional role of *H19* in the osteogenic and adipogenic differentiation of hBMSCs in the ONFH group, siRNAs were used to silence *H19*, followed by osteogenic and adipogenic induction. qRT-PCR analysis confirmed that *H19* expression was significantly reduced following siRNA-mediated knockdown (Fig 4A). Silencing of *H19* significantly inhibited osteogenic differentiation, as evidenced by a decrease in mineralized nodule formation (Fig 4B and C) and a reduction in the protein levels of RUNX2 and COL1A1 following osteogenic treatment (Fig 4F and G). Additionally, *H19* knockdown markedly promoted adipogenic differentiation, as indicated by an increase in the number of differentiated adipocytes (Fig 4D and E) and elevated protein levels of FABP4 and PPARγ following adipogenic treatment (Fig 4F and H).

Subsequently, the effects of *H19* overexpression on osteoblast and adipocyte differentiation were examined. qRT-PCR analysis confirmed successful overexpression of *H19* (Fig 4I). In contrast to the results observed with *H19* knockdown, *H19* overexpression significantly increased mineralized nodule formation (Fig 4J and K) and elevated the protein levels of RUNX2 and COL1A1 (Fig 4N and O). Moreover, *H19* upregulation markedly inhibited adipogenic differentiation, as evidenced by a reduction in the number of differentiated adipocytes (Fig 4L and M) and decreased protein levels of FABP4 and PPARγ following adipogenic treatment (Fig 4N and P).

Collectively, these findings indicate that *H19* silencing inhibits osteoblast differentiation and promotes adipocyte differentiation in hBMSCs of the ONFH group, whereas *H19* overexpression facilitates osteoblast differentiation and suppresses adipocyte differentiation.

### H19-Mediated Pro-Osteogenic and Anti-Adipogenic Effects Are Associated with Activation of the Wnt/β-Catenin Signaling Pathway

**Fig 5:**
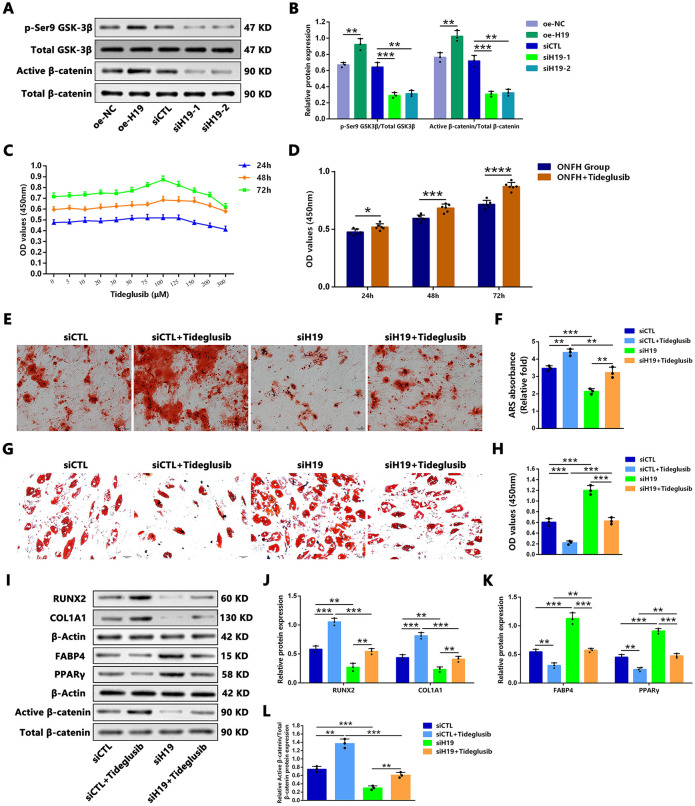
H19-mediated pro-osteogenic and anti-adipogenic effects were associated with the activation of the Wnt/β-catenin signaling pathway. **(A–B)** Representative western blot image (A) and quantitative analysis (B) indicated the effects of *H19* overexpression and silencing in hBMSCs of the ONFH group on the protein expressions of p-Ser9 *GSK-3β*, and active β-catenin (n = 3). **(C)** When the concentration of tideglusib reached 100 μM, cellular viability reached its peak at 24, 48, and 72 h (n = 6). **(D)** Cellular proliferative capacity was assessed at 24, 48, and 72 h after cells were treated with tideglusib (n = 6). **(E–F)** ARS staining (E) and quantification (F) showed the effects of *H19* silencing with or without tideglusib treatment on the calcium deposits in hBMSCs (n = 3). Scale bars: 100 μm. **(G–H)** ORO staining (G) and quantification (H) showed the effects of *H19* silencing with or without tideglusib treatment on the intracellular lipid accumulation in hBMSCs (n = 3). Scale bars: 50 μm. **(I–L)** After hBMSCs of *H19* silencing with or without tideglusib treatment, the representative western blot image (I) and quantitative analysis showed the protein levels of RUNX2 and COL1A1 relative to β-actin **(J)**, FABP4 and PPARγ relative to β-actin **(K)**, and active β-catenin relative to total β-catenin **(L)** (n = 3). (B,F,H,J,K,L) Statistical analysis was performed using one/two-way ANOVA with Bonferroni’s post-hoc test. Data are presented as mean ± SD, n = 3 per group, #P > 0.05, **P < 0.01, ***P < 0.001. **(D)** Data are presented as mean ± SD, n = 3 per group, #P > 0.05, **P < 0.01, ***P < 0.001, unpaired Student’s t test.

GCs have been demonstrated to activate *GSK-3β* through the dephosphorylation of Ser9 of *GSK-3β*[33, 34], thereby inducing the degradation of cytoplasmic β-catenin and inactivating the Wnt/β-catenin signaling pathway, which plays a pivotal role in the impaired osteogenesis and enhanced adipogenesis of MSCs following GC administration [11,16,35–37]. Therefore, the involvement of the Wnt/β-catenin signaling pathway in the pro-osteogenic and anti-adipogenic effects of *H19* in hBMSCs of GC-induced ONFH patients was further investigated. Western blot analysis was used to assess the relative protein levels of phosphorylated (Ser9) *GSK-3β* (p-Ser9 *GSK-3β*) and non-phosphorylated (active) β-catenin in hBMSCs with *H19* overexpression or silencing. The results demonstrated that *H19* upregulation increased the relative protein levels of p-Ser9 *GSK-3β* and active β-catenin, whereas *H19* downregulation led to decreased relative protein levels of these proteins (Fig 5A and B).

To further examine whether osteogenic and adipogenic differentiation capacities were altered following *H19* silencing in hBMSCs treated with a *GSK-3β* inhibitor, the CCK-8 assay was performed to determine the effects of the *GSK-3β* inhibitor tideglusib on cell viability in ONFH group hBMSCs. Cellular viability peaked at a concentration of 100 μM following 24, 48, and 72 h of tideglusib treatment (Fig 5C and D). Thus, 100 μM was selected for subsequent experiments.

On the one hand, mineralized nodule formation and the protein levels of RUNX2 and COL1A1 in *H19*-silenced hBMSCs treated with tideglusib were higher than in untreated *H19*-silenced cells but remained lower than those observed in siCTL group hBMSCs treated with tideglusib (Fig 5E, F, I, and J). On the other hand, the number of ORO-positive adipocytes and the protein levels of FABP4 and PPARγ in *H19*-silenced hBMSCs treated with tideglusib were lower than in untreated *H19*-silenced cells but remained higher than those in siCTL group hBMSCs treated with tideglusib (Fig 5G, H, I, and K). Additionally, the relative protein levels of active β-catenin in *H19*-silenced hBMSCs treated with tideglusib were significantly increased compared with untreated *H19*-silenced cells, though still lower than in siCTL group hBMSCs treated with tideglusib (Fig 5I and L).

In summary, overexpression of *H19* in hBMSCs from GC-induced ONFH patients decreased the phosphorylation of β-catenin by *GSK-3β*, thereby preventing β-catenin degradation. Conversely, *H19* silencing increased β-catenin phosphorylation by *GSK-3β*, thereby promoting β-catenin degradation, whereas the *GSK-3β* inhibitor tideglusib partially rescued the impaired osteogenesis and enhanced adipogenesis induced by *H19* silencing.

### Cellular therapy with Dnmt1-Knockdown rBMSCs Promotes Bone Repair and Reduces Fat Accumulation in a GC-Induced ONFH Rat Model

**Fig 6:**
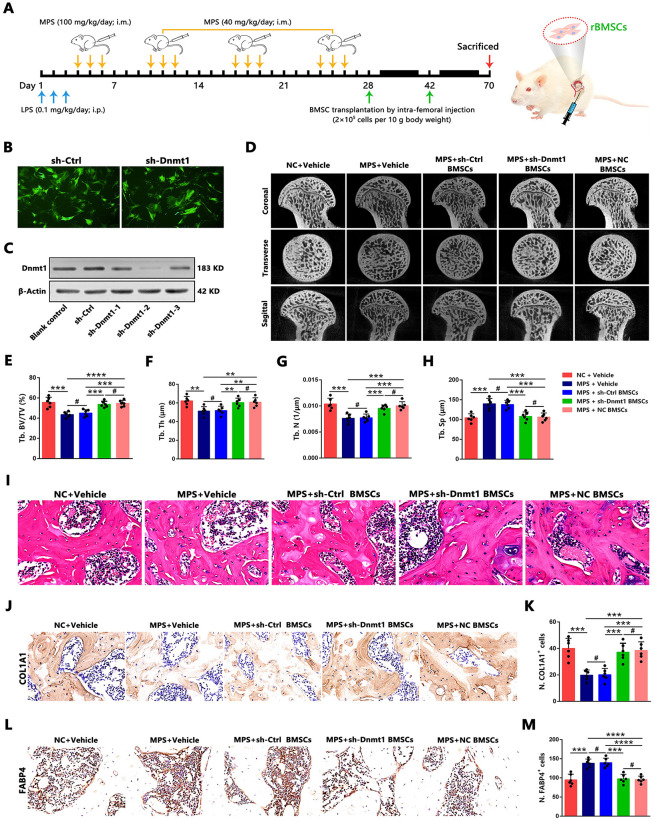
Dnmt1 knockdown BMSC implantation promoted bone repair and reduced fat accumulation in ONFH rat model. **(A)** Schematic diagram of the experimental design for investigating the effects of implantation with *Dnmt1*-knockdown or *H19*-overexpression rBMSCs on the femoral heads of MPS-treated rats. **(B)** Fluorescence microscopy was used to observe infection efficiency in primary rBMSCs. Scale bars: 100 μm. **(C)** Western blot analysis showed that *Dnmt1* shRNA efficiently decreased the protein expression levels of *Dnmt1* in rBMSCs of MPS-treated rats. β-actin was used as a loading control. **(D)** Representative micro-CT images of the femoral head of each treatment group at week 6 after rBMSC transplantation are shown. **(E–H)** Micro-CT quantitative results are expressed as BV/TV **(E)**, Tb.Th **(F)**, Tb.N **(G)**, and Tb.Sp **(H)** (n = 6). **(I)** Representative images of H&E staining for each treatment group at week 6 after rBMSC implantation were shown and osteonecrosis was characterized by the empty lacunae (black arrow) or pyknotic nucleus of osteocytes (blue arrow) in trabecular bone. Scale bars: 25 μm. **(J–K)** Representative IHC images and quantitative analysis of positive stain of COL1A1 in the femoral head of each treatment group were shown (n = 6). Scale bars: 25 μm. **(L–M)** Representative IHC images and quantitative analysis of positive stain of FABP4 in the femoral head of each treatment group are shown (n = 6). Scale bars: 25 μm. Statistical analysis was performed using one/two-way ANOVA with Bonferroni’s post-hoc test. Data are presented as mean ± SD, #P > 0.05, **P < 0.01, ***P < 0.001, ****P < 0.0001. NC: normal control, sh/shRNA: short hairpin RNA, sh-Ctrl: control shRNA.

Given the present findings that *Dnmt1* knockdown in hBMSCs from the ONFH group enhances osteogenesis and attenuates adipogenesis, it was hypothesized that implantation of *Dnmt1*-knockdown BMSCs derived from GC-induced ONFH might mitigate disease progression by restoring the osteo-adipogenic balance. A rat model of GC-induced ONFH was established using a combination of LPS and MPS, as previously described (see Materials and Methods) and illustrated in Fig 6A. Intra-femoral injections were performed at weeks 4 and 6 following the induction of GC-induced ONFH. After completing the four-week MPS injections, primary rBMSCs were isolated and cultured as described above. These cells were then stably transfected with either a lentiviral empty vector or a lentiviral vector targeting *Dnmt1* for knockdown. The efficiency of *Dnmt1* knockdown was confirmed by fluorescence microscopy, showing equivalent infection rates in the control non-targeting shRNA (sh-Ctrl) group and the shRNA targeting *Dnmt1* (sh-*Dnmt1*) group (Fig 6B). Additionally, western blot analysis verified the efficiency of *Dnmt1* knockdown (Fig 6C).

The therapeutic efficacy of rBMSC transplantation was assessed through micro-CT analysis, H&E staining, and IHC analysis. IHC staining at week 6 post-injection revealed a widespread and uneven distribution of BrdU-positive cells in the cartilage, subchondral area, and bone marrow cavity of the femoral head (S3 Fig). Micro-CT imaging demonstrated that the MPS group exhibited a significant reduction in trabecular bone mass and extensive destruction of the trabecular structure in the femoral head, characterized by sparse trabecular bone and subchondral cystic degeneration, compared with the NC group (Fig 6D). Furthermore, quantitative analysis of micro-CT data revealed that MPS injection resulted in significant deterioration of trabecular bone parameters, including markedly reduced BV/TV, Tb.Th, and Tb.N, as well as significantly increased Tb.Sp, in comparison with the NC group (Fig 6E-H). Notably, structural integrity and trabecular bone microstructural parameters were significantly improved in MPS-treated rats implanted with either *Dnmt1*-knockdown rBMSCs (sh-*Dnmt1* rBMSCs) or NC rBMSCs, whereas no significant improvements were observed in MPS-treated rats receiving control non-targeting shRNA rBMSCs (sh-Ctrl rBMSCs) (Fig 6D-H).

Histological analysis using H&E staining revealed diffuse empty lacunae and pyknotic nuclei of osteocytes in trabecular bone in the MPS group (Fig 6I), consistent with established criteria for osteonecrosis [49]. Cellular therapy via intra-femoral injection with either sh-*Dnmt1* rBMSCs or NC rBMSCs significantly reduced the presence of empty lacunae and pyknotic nuclei of osteocytes in trabecular bone, whereas no significant reduction was observed in MPS-treated rats implanted with sh-Ctrl rBMSCs (Fig 6I). To further assess the effects of rBMSC transplantation on osteoblastic bone formation and bone marrow fat accumulation in the femoral head of the GC-induced ONFH rat model, IHC staining was employed to detect the expression of the osteogenic marker COL1A1 and the adipogenic marker FABP4. IHC staining results demonstrated that, compared with the NC group, COL1A1 expression was decreased while FABP4 expression was increased in both the MPS group and the MPS-treated rats implanted with sh-Ctrl rBMSCs, indicating impaired osteogenesis and enhanced adipogenesis in these groups (Fig 6J-M). Moreover, compared with the MPS group, COL1A1 expression was significantly elevated while FABP4 expression was markedly reduced in MPS-treated rats implanted with either sh-*Dnmt1* rBMSCs or NC rBMSCs (Fig 6J-M).

Taken together, transplantation of genetically modified *Dnmt1*-knockdown rBMSCs via intra-femoral injection promoted bone repair and reduced fat accumulation in the GC-induced ONFH rat model, thereby effectively delaying disease progression.

### Cellular therapy with H19-Overexpressing rBMSCs promotes bone repair and reduces fat accumulation in a GC-Induced ONFH Rat Model

**Fig 7:**
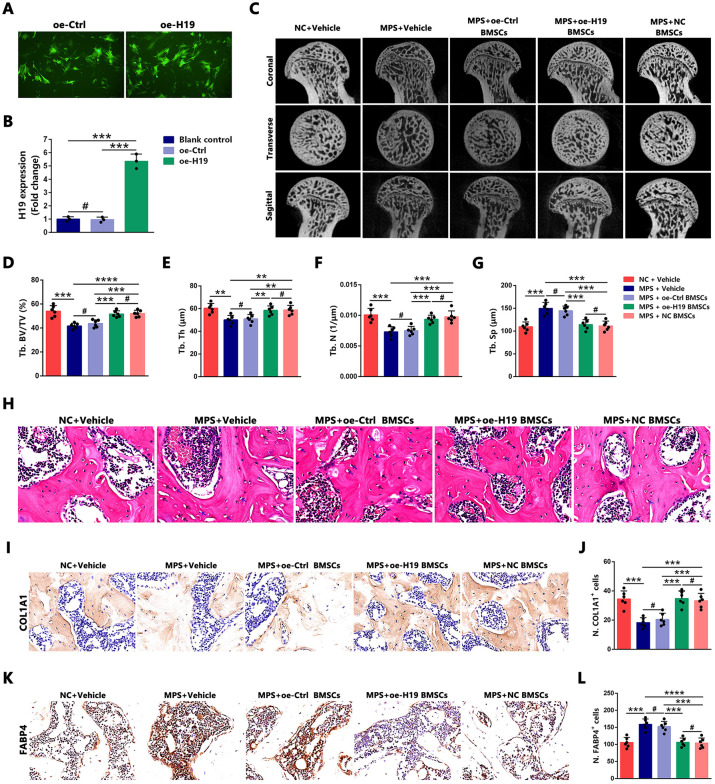
H19 overexpression BMSC implantation promoted bone repair and reduced fat accumulation in the ONFH rat model. **(A)** Fluorescence microscopy was used to observe infection efficiency in primary rBMSCs of MPS-treated rats. Scale bars: 100 μm. (B) qRT-PCR analysis verified the overexpression of *H19* in rBMSCs of MPS-treated rats (n = 3). **(C)** Representative micro-CT images of the femoral head of each treatment group at week 6 after BMSC transplantation are presented. **(D–G)** Micro-CT quantitative results are expressed as BV/TV **(D)**, Tb.Th **(E)**, Tb.N **(F)**, and Tb.Sp **(G)** (n = 6). **(H)** Representative images of H&E staining for each treatment group at week 6 after rBMSC implantation are shown, and osteonecrosis was characterized by the empty lacunae (black arrow) or pyknotic nucleus of osteocytes (blue arrow) in trabecular bone. Scale bars: 25 μm. **(I–J)** Representative IHC images and quantitative analysis of positive stain of COL1A1 in the femoral head of each treatment group are presented (n = 6). Scale bars: 25 μm. **(K–L)** Representative IHC images and quantitative analysis of positive stain of FABP4 in the femoral head of each treatment group are shown (n = 6). Scale bars: 25 μm. Statistical analysis was performed using one/two-way ANOVA with Bonferroni’s post-hoc test. Data are presented as mean ± SD, #P > 0.05, **P < 0.01, ***P < 0.001, ****P < 0.0001. NC: normal control, oe: overexpression, oe-Ctrl: empty vector control for overexpression.

Given that *H19* overexpression was also found to enhance osteogenic differentiation and suppress adipogenic differentiation in hBMSCs, further investigation was conducted to determine whether intra-femoral injection of *H19*-overexpressing rBMSCs could ameliorate the osteo-adipogenic imbalance and consequently delay the progression of GC-induced ONFH. The procedural details for cellular therapy with *H19*-overexpressing rBMSCs were identical to those employed for *Dnmt1*-knockdown rBMSCs (Fig 6A). Stable *H19*-overexpressing cell lines were generated via lentiviral transfection, and fluorescence microscopy confirmed equal infection efficiency between the lentiviral empty vector (oe-Ctrl) group and the *H19*-overexpression lentiviral vector (oe-*H19*) group (Fig 7A). Furthermore, qRT-PCR analysis demonstrated a significant increase in *H19* expression in cells transfected with the *H19* lentiviral vector (Fig 7B).

Findings from micro-CT analysis, H&E staining, and IHC analysis indicated that intra-femoral injection of *H19*-overexpressing rBMSCs exerted therapeutic effects similar to those observed with *Dnmt1*-knockdown rBMSCs. Specifically, micro-CT analysis revealed that deterioration of trabecular structure and trabecular bone parameters was markedly reversed in MPS-treated rats implanted with either *H19*-overexpressing rBMSCs (oe-*H19* rBMSCs) or NC rBMSCs, whereas no significant improvements were observed in MPS-treated rats receiving rBMSCs transfected with an empty vector-expressing lentivirus (Fig 7C-G). Histological analysis using H&E staining demonstrated that, compared with the MPS group, the number of empty lacunae and pyknotic nuclei of osteocytes in trabecular bone was significantly reduced in MPS-treated rats implanted with either oe-*H19* rBMSCs or NC rBMSCs, whereas no significant reduction was observed in MPS-treated rats implanted with oe-Ctrl rBMSCs (Fig 7H). Additionally, IHC staining analysis indicated that the osteo-adipogenic imbalance in the femoral head of rats was significantly alleviated following intra-femoral injection with either oe-*H19* rBMSCs or NC rBMSCs (Fig 7I-L).

Collectively, implantation of *H19*-overexpressing rBMSCs enhanced bone repair and reduced fat accumulation in the GC-induced ONFH rat model, thereby effectively delaying disease progression.

## Discussion

Alterations in DNA methylation levels and patterns in the promoter region of the *H19* gene have been observed in various osteogenic and bone-related diseases. In calcific aortic valve disease, hypomethylation of the *H19* promoter and increased *H19* expression, which exert a strong pro-osteogenic effect, have been closely associated with rapid disease progression [26]. Furthermore, mechanical unloading has been shown to induce hypermethylation of the *H19* promoter, leading to *H19* downregulation and insufficient osteogenic capacity, as observed in disuse osteoporosis [25]. However, most of these studies were conducted in non-GC-associated bone diseases. Our study is the first to demonstrate that GC exposure induces hypomethylation of the *H19* promoter and its subsequent overexpression in undifferentiated BMSCs from ONFH patients, which contrasts with the hypermethylation observed in other conditions. Moreover, we found that *H19* expression decreases upon osteogenic or adipogenic differentiation in GC-treated BMSCs, suggesting a differentiation-stage-specific regulatory mechanism that is unique to GC-induced ONFH. These findings suggest that DNA methylation in the *H19* promoter plays a crucial role in the regulation of osteogenesis and bone metabolism. However, whether GC-induced ONFH is associated with aberrant DNA methylation at the *H19* promoter and dysregulated *H19* expression in hBMSCs remains unclear. In the present study, we observed a notable phenomenon: DNA hypomethylation of the *H19* promoter resulted in *H19* overexpression in undifferentiated hBMSCs from GC-induced ONFH patients, whereas decreased *H19* expression was detected in these cells following osteogenic or adipogenic differentiation. Additionally, *Dnmt1* expression was negatively correlated with *H19* in both undifferentiated and differentiated BMSCs. Furthermore, the nuclear localization and reduced expression of *Dnmt1*, accompanied by the increased nuclear distribution of *H19* in undifferentiated hBMSCs from GC-induced ONFH patients, suggest that exposure of hBMSCs to GCs may induce the translocation of *H19* from the cytoplasm to the nucleus. The underlying mechanism of this phenomenon remains unclear. Previous studies have indicated that the GC-induced loss of DNA methylation is closely related to cell division [50]. A plausible explanation is that GCs induce the loss of DNA methylation in BMSCs, whereas the DNA methylation status and the ability of GCs to induce DNA methylation loss are altered during osteogenic and adipogenic differentiation of BMSCs.

It has been reported that all three types of *DNMTs* play significant yet distinct roles in osteogenesis and bone metabolism under various physiological and pathological conditions [51–53]. Our knockdown experiments demonstrated that only *Dnmt1* knockdown in hBMSCs from GC-induced ONFH patients, but not *Dnmt3a* or *Dnmt3b* knockdown, contributed to increased *H19* expression, enhanced osteogenic differentiation, and attenuated adipogenic differentiation. Conversely, *Dnmt1* overexpression exhibited the opposite effects. These findings indicate that *Dnmt1* is the primary DNMT responsible for the epigenetic regulation of *H19* expression, as well as for the modulation of osteogenesis and adipogenesis in hBMSCs from GC-induced ONFH patients. Furthermore, our gene silencing and overexpression experiments demonstrated that *H19* promotes osteogenesis while inhibiting adipogenesis in hBMSCs from GC-induced ONFH patients, consistent with previous studies [22,23].

Previous research has shown that GCs activate *GSK-3β* by dephosphorylation at the Ser9 site [34,35], thereby inhibiting β-catenin nuclear translocation and the subsequent transcription of Wnt target genes, including those involved in osteogenesis and adipogenesis [11,16,36–38]. Additionally, GC-induced activation of *GSK-3β* has been implicated in the apoptosis of osteoblasts [54]. In the present study, we found that *H19* inhibits *GSK-3β* activity by promoting its phosphorylation at the Ser9 site. The inactivation of *GSK-3β* suppresses β-catenin degradation, thereby activating Wnt/β-catenin signaling. Tideglusib, a selective and irreversible small-molecule non-ATP-competitive *GSK-3β* inhibitor, has demonstrated good efficacy, safety, and tolerability in the treatment of Alzheimer’s disease, as established in a phase II trial [55]. Moreover, tideglusib has been reported to promote dentin formation and bone regeneration [56–58]. In our study, the reduction in active β-catenin expression, along with impaired osteogenesis and enhanced adipogenesis caused by *H19* silencing, was partially rescued by tideglusib treatment. These findings suggest that *H19* promotes osteogenic differentiation while inhibiting adipogenic differentiation in hBMSCs from GC-induced ONFH patients by suppressing *GSK-3β* activity, thereby reducing β-catenin degradation.

Currently, core decompression (CD) combined with BMSC transplantation has garnered considerable attention as a treatment for early-stage ONFH, demonstrating efficacy in alleviating pain, improving hip function, and reducing reoperation rates [59,60]. However, the long-term effectiveness of CD combined with BMSC transplantation remains debatable. The low cellular viability and diminished osteogenic differentiation capacity of autologous MSCs may contribute to treatment failure and disease progression in GC-induced ONFH patients [17]. Although allogeneic MSC transplantation has shown beneficial effects in early-stage ONFH [61,62], limitations such as donor availability, immunogenicity, and ethical concerns hinder its clinical application. Additionally, previous studies have demonstrated that the implantation of genetically modified BMSCs significantly enhances bone repair in osteonecrotic lesions during early-stage GC-induced ONFH [63,64]. Therefore, identifying an effective strategy to restore the osteogenic differentiation potential of BMSCs in GC-induced ONFH patients is critical for the success of autologous stem cell therapy. In the present study, we first isolated BMSCs from rats following a four-week MPS induction and subsequently generated genetically modified BMSCs using lentiviral vectors carrying the rat *Dnmt1* or *H19* gene. The results demonstrated that the intrafemoral injection of genetically modified *Dnmt1*-knockdown or *H19*-overexpressing BMSCs in a GC-induced ONFH rat model significantly reduced empty lacunae and improved the osteo-adirogenic imbalance, thereby mitigating disease progression.

Several important considerations arise from the imprinted nature of the *H19/IGF2* locus. First, our bisulfite sequencing and expression analyses measured the bulk methylation status and total *H19* RNA levels across the cell population and therefore do not distinguish between allelic contributions. While genomic imprinting is generally stable in somatic cells, it can be dysregulated in disease states [65,66]. Whether GC exposure alters the imprinting status (e.g., induces loss of imprinting) in BMSCs remains an open question that warrants future investigation using allele-specific assays. Second, as *H19* and *IGF2* expression are reciprocally regulated due to their shared ICR, the observed *H19* hypomethylation and overexpression in undifferentiated ONFH BMSCs might be accompanied by dysregulated *IGF2* expression. *IGF2* is a potent growth factor known to influence bone metabolism and MSC differentiation [67,68]. Therefore, the phenotypic effects we attribute to *H19* could potentially involve altered *IGF2* signaling. Future studies should quantify *IGF2* expression in this model to fully understand the regulatory dynamics at this locus.

From a therapeutic perspective, the use of *H19*-overexpressing BMSCs necessitates careful consideration. Forced overexpression via lentiviral vectors likely bypasses the endogenous imprinted regulation, potentially leading to supraphysiological *H19* levels that could have unforeseen consequences, including perturbing the *H19/IGF2* balance. While our in vivo results showed a beneficial effect on delaying ONFH progression, the long-term safety and stability of such an approach require further evaluation to ensure that the therapeutic manipulation does not inadvertently promote oncogenic transformation or other adverse effects, as the *H19/IGF2* locus has been implicated in various cancers [21].

Furthermore, our methodological approach to assessing DNA methylation warrants discussion. The cloning and sequencing of bisulfite-converted PCR products, while a standard technique, has inherent limitations when applied to imprinted loci. For a typically heterogeneously methylated promoter, sequencing multiple clones is essential to capture the full spectrum of methylation patterns within a cell population. This is particularly critical for the *H19* promoter, which is characterized by its imprinted state—expected to be fully methylated on one parental allele and completely unmethylated on the other. Our analysis, which sequenced multiple clones per sample and reported the average methylation, would be expected to yield a value around 50% in a normal, imprinted cell population. The significantly lower average methylation we observed in undifferentiated ONFH BMSCs (~20–30%, Fig. 1D-E) strongly suggests a pathological shift from this balanced imprinted state towards widespread hypomethylation, potentially affecting a subset of alleles. This finding of aberrant hypomethylation, rather than a loss of the absolute dichotomous methylated/unmethylated state, highlights a disease-specific epigenetic dysregulation at this locus. Future studies employing techniques such as next-generation bisulfite sequencing could provide single-molecule resolution to precisely characterize the distribution of methylation patterns and definitively assess allelic contributions in this context.

Another important consideration in interpreting our DNA methylation data is the potential influence of confounding variables. Factors such as age, sex, genetics, underlying comorbidities, and the specific GC treatment regimen (dose and duration) are known to influence the epigenome. In our patient cohort, we matched the ONFH and control (FNF) groups for age and sex to mitigate these sources of bias (Table 1). However, the ONFH group had a significantly higher BMI, which is a known clinical correlate of GC-induced ONFH and could itself be associated with epigenetic alterations. While it is challenging to completely disentangle the effects of GC exposure from associated metabolic factors like higher BMI, our primary in vitro model—where BMSCs from patients were cultured and treated with GCs or 5-aza-dC—provides strong evidence for a direct, cell-autonomous effect of GCs on *Dnmt1* expression and *H19* promoter methylation. Furthermore, the consistent rescue of phenotype by 5-aza-dC and genetic manipulation (knockdown/overexpression) in these cells supports a causal role for this epigenetic axis. Nonetheless, we acknowledge that patient-specific genetic backgrounds and other unmeasured variables may contribute to inter-individual variation in methylation patterns, a common challenge in human epigenetic studies. Future investigations with larger, prospectively recruited cohorts could help stratify these influences and further validate the robustness of our findings.

A potential limitation of our clinical cohort analysis is the unmatched BMI between ONFH and control groups. Higher BMI is a well-documented clinical correlate and risk factor for steroid-induced ONFH, often reflecting the underlying disease state or metabolic effects of glucocorticoid treatment itself. Therefore, this difference is an expected characteristic of the disease population rather than a random confounding variable. While we cannot entirely rule out that metabolic factors associated with higher BMI may independently influence the BMSC epigenome, our core mechanistic findings are robust against this concern. The in vitro GC exposure, pharmacological demethylation, and genetic rescue experiments were performed on isolated BMSCs, effectively disentangling the direct cellular effects of glucocorticoids from systemic metabolic influences. Consequently, the identified Dnmt1/H19/GSK-3βpathway is supported as a direct target of glucocorticoid action in BMSCs, with BMI representing a relevant clinical comorbidity rather than a driver of the observed epigenetic dysregulation.

The potential influence of cell passage number on DNA methylation and differentiation capacity is a well-known consideration in MSC research [69,70]. To mitigate this confounding variable, we standardized our experiments by using low-passage (P2-P3) hBMSCs for all in vitro manipulations, including treatments, transfections, and differentiation assays. This approach helps to maintain a more stable epigenetic state and reduces the variability associated with long-term culture. While we did not systematically test the effect of passage number across a wide range within this study, the use of a narrow, low-passage window for all experiments strengthens the internal validity of our comparisons between treatment groups. Future studies could explicitly investigate how prolonged in vitro expansion influences the GC-*Dnmt1*-*H19* pathway described here.

A critical consideration for the translational relevance of our rat model is the comparability of the GC dosing regimen to human clinical scenarios. The rat model employed a high-dose pulse of methylprednisolone (MPS; 100 mg/kg for 3 days) followed by a lower maintenance dose (40 mg/kg, 3 times/week for 3 weeks). While this dose is high on a per-weight basis, it is a well-established and widely used protocol for efficiently inducing osteonecrosis in rodents, reflecting a scenario of intense, pulsed GC therapy [44]. When adjusted for body surface area using standard conversion factors, this regimen approximates the high-end cumulative doses associated with a significant risk of developing ONFH in patients receiving pulse steroid therapy for conditions like severe autoimmune disorders or post-transplant immunosuppression [71,72]. Therefore, the model recapitulates the pathophysiological effects of high-dose GC exposure relevant to the human condition, rather than mimicking a precise mg-for-kg equivalent dose.

## Conclusions

**Fig 8:**
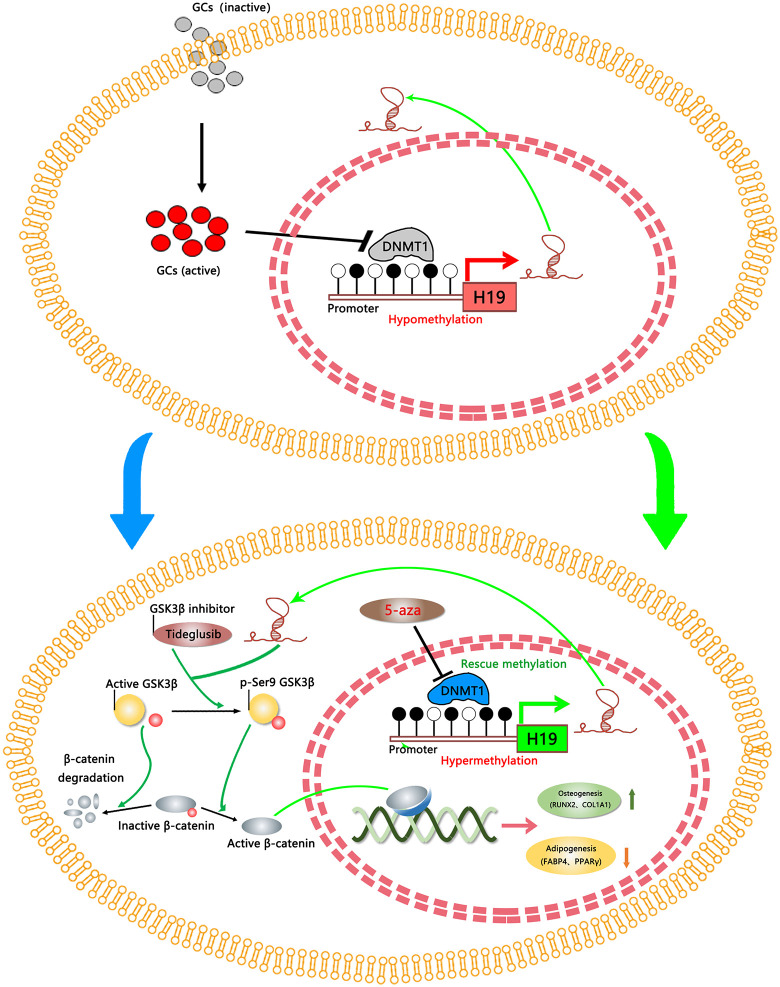
A schematic diagram summarizing the proposed role of the Dnmt1/H19/GSK-3β axis in the pathogenesis of GC-induced ONFH.

In summary, our findings demonstrate that *H19* and *Dnmt1* expression levels are negatively correlated in both undifferentiated and osteo-adipogenically differentiated hBMSCs. Among the three *DNMTs*, *Dnmt1* plays a predominant role in the epigenetic regulation of *H19* expression and serves as a reciprocal modulator of osteogenic and adipogenic differentiation in hBMSCs from GC-induced ONFH patients. Conversely, *H19* exhibits an opposing modulatory effect on osteoblast and adipocyte differentiation. The function of *H19* is mediated through its inhibition of *GSK-3β* activity, thereby reducing β-catenin degradation and activating the Wnt/β-catenin signaling pathway. Furthermore, *in vivo* studies demonstrated that the implantation of *Dnmt1*-knockdown or *H19*-overexpressing rBMSCs in an early-stage GC-induced ONFH rat model significantly reduced empty lacunae and improved the osteo-adipogenic imbalance, thereby ameliorating disease progression. A schematic diagram summarizing the proposed role of the Dnmt1/*H19*/GSK-3β axis in the pathogenesis of GC-induced ONFH is presented in Fig 8. Collectively, our findings provide novel insights into the role of DNA methylation alterations in the *H19* promoter in the regulation of osteogenic and adipogenic differentiation in hBMSCs from GC-induced ONFH patients and identify potential therapeutic targets for the prevention and treatment of GC-induced ONFH.

## Supporting information

S1 Fig The subcellular localization of Dnmt1 and H19 in undifferentiated hBMSCs of the control group and ONFH group. (A) The localization and protein expression of *Dnmt1* in hBMSCs from the ONFH and control groups were detected by immunofluorescence. Scale bars: 50 μm. (B) The relative distribution of *H19* in subcellular fractions was detected by qRT-PCR analyses of fractionated nuclear and cytoplasmic RNA. The graph depicts the nuclear-to-cytoplasmic (N/C) ratio of *H19* expression, calculated after normalization to U6 (nuclear marker) and GAPDH (cytoplasmic marker), respectively. Data are presented as mean ± SD, **P < 0.01, unpaired Student’s t-test.
(TIF)

S2 Fig 5-aza-dC enhanced osteogenic differentiation and inhibited adipogenic differentiation of hBMSCs from the ONFH group. (A–B) qRT-PCR analysis revealed the relative expression of osteogenic marker genes, RUNX2 (A) and COL1A1 (B), in hBMSCs at days 3, 7, and 14 during osteogenic differentiation (n = 10). (C–D) qRT-PCR analysis showed the relative expression of adipogenic marker genes, FABP4 (C) and PPARγ (D), in hBMSCs at days 3, 7, and 14 during adipogenic differentiation (n = 10). (E–F) ARS staining (E) and quantification (F) were performed to measure the calcium deposits for matrix mineralization in hBMSCs after 14 days of osteogenic induction (n = 6). Scale bars: 100 μm. (G–H) ORO staining (G) and quantification (H) were used to evaluate intracellular lipid accumulation in hBMSCs after 21 days of adipogenic induction (n = 6). Scale bars: 50 μm. Statistical analysis was performed using one/two-way ANOVA with Bonferroni’s post-hoc test. Data are presented as mean ± SD, *P < 0.05, **P < 0.01, ***P < 0.001, ****P < 0.0001.
(TIF)

S3 Fig IHC staining of BrdU at week 6 after rBMSC transplantation. Scale bars: 50 μm.
(TIF)

S1 Table Detailed demographic and clinical characteristics of each patient in the control group and the ONFH group. (DOCX)

S2 Table Primer sequences used for qRT-PCR quantifications. (DOCX)

S3 Table siRNA sequences used for gene silencing. (DOCX)

S4 Table shRNA sequences used for gene silencing. (DOCX)

S5 Table The expression levels of DNMTs, H19, and osteogenic markers in MSCs during osteogenic differentiation in Group S versus Group C were assessed using quantitative real-time PCR (qRT-PCR). (XLSX)

S6 Table The expression levels of DNMTs, H19, and adipogenic markers in MSCs during adipogenic differentiation in Group S versus Group C were assessed using quantitative real-time PCR (qRT-PCR). (XLSX)

S7 Table The expression levels of DNMTs and H19 in MSCs from Group S compared to Group C prior to differentiation were measured using quantitative real-time PCR (qRT-PCR). (XLSX)

S1 File Uncropped scans of Western blots in manuscript. (ZIP)

S2 File Raw data of Bisulfite sequencing. (ZIP)
