# Supplementary figures and images for: Glucocorticoid-Induced alterations in DNA methylation in the H19 promoter of Bone Marrow-Derived Mesenchymal Stem Cells are associated with the pathogenesis of osteonecrosis
Source: PLoS One. 2026 Mar 27;21(3):e0345372. doi: 10.1371/journal.pone.0345372 (PMC13028513; doi:10.1371/journal.pone.0345372)

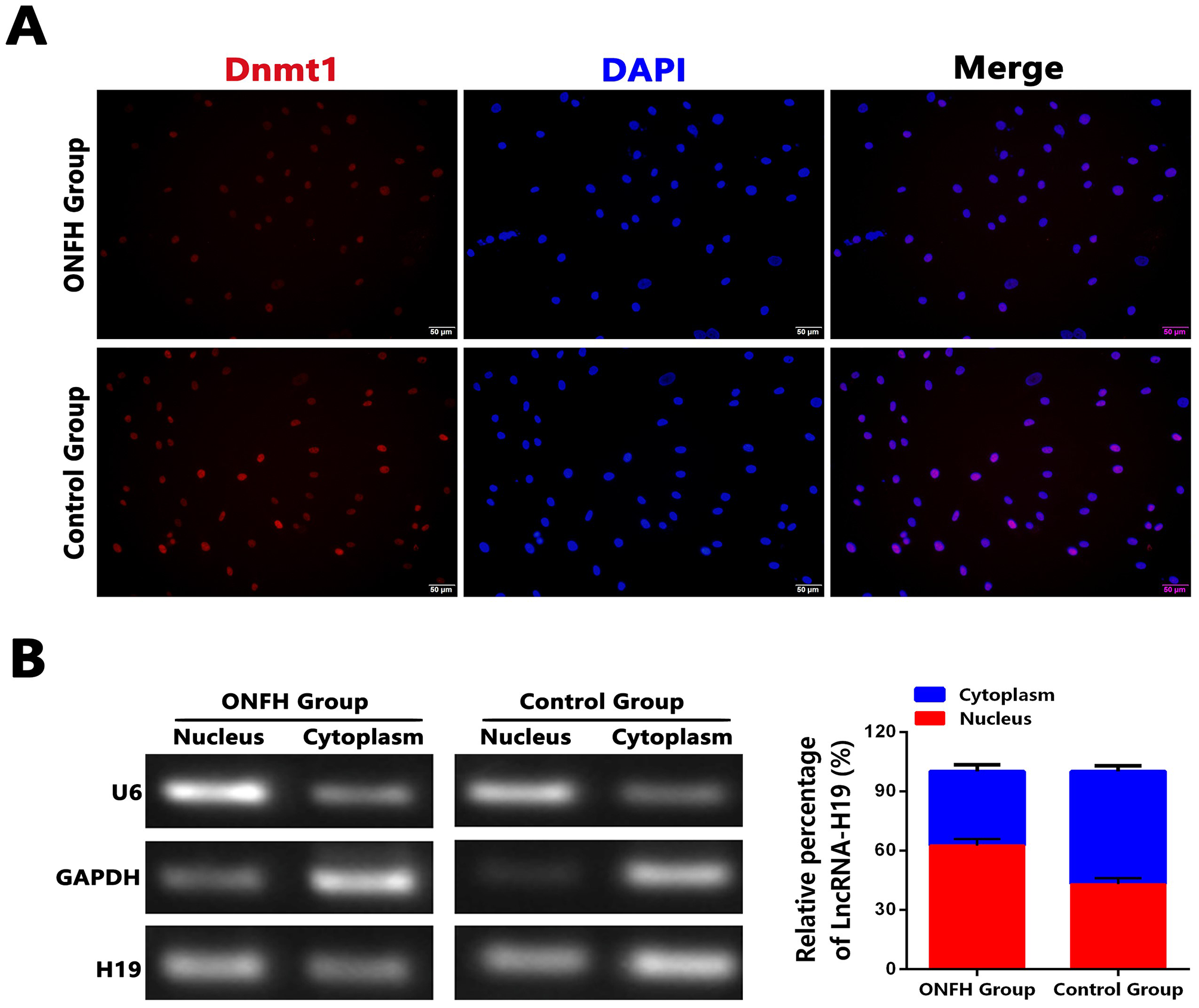

Supplement: S1 Fig — (A) The localization and protein expression of Dnmt1 in hBMSCs from the ONFH and control groups were detected by immunofluorescence. Scale bars: 50 μm. (B) The relative distribution of H19 in subcellular fractions was detected by qRT-PCR analyses of fractionated nuclear and cytoplasmic RNA. The graph depicts the nuclear-to-cytoplasmic (N/C) ratio of H19 expression, calculated after normalization to U6 (nuclear marker) and GAPDH (cytoplasmic marker), respectively. Data are presented as mean ± SD, **P < 0.01, unpaired Student’s t-test. (TIF) [file pone.0345372.s001.tif]

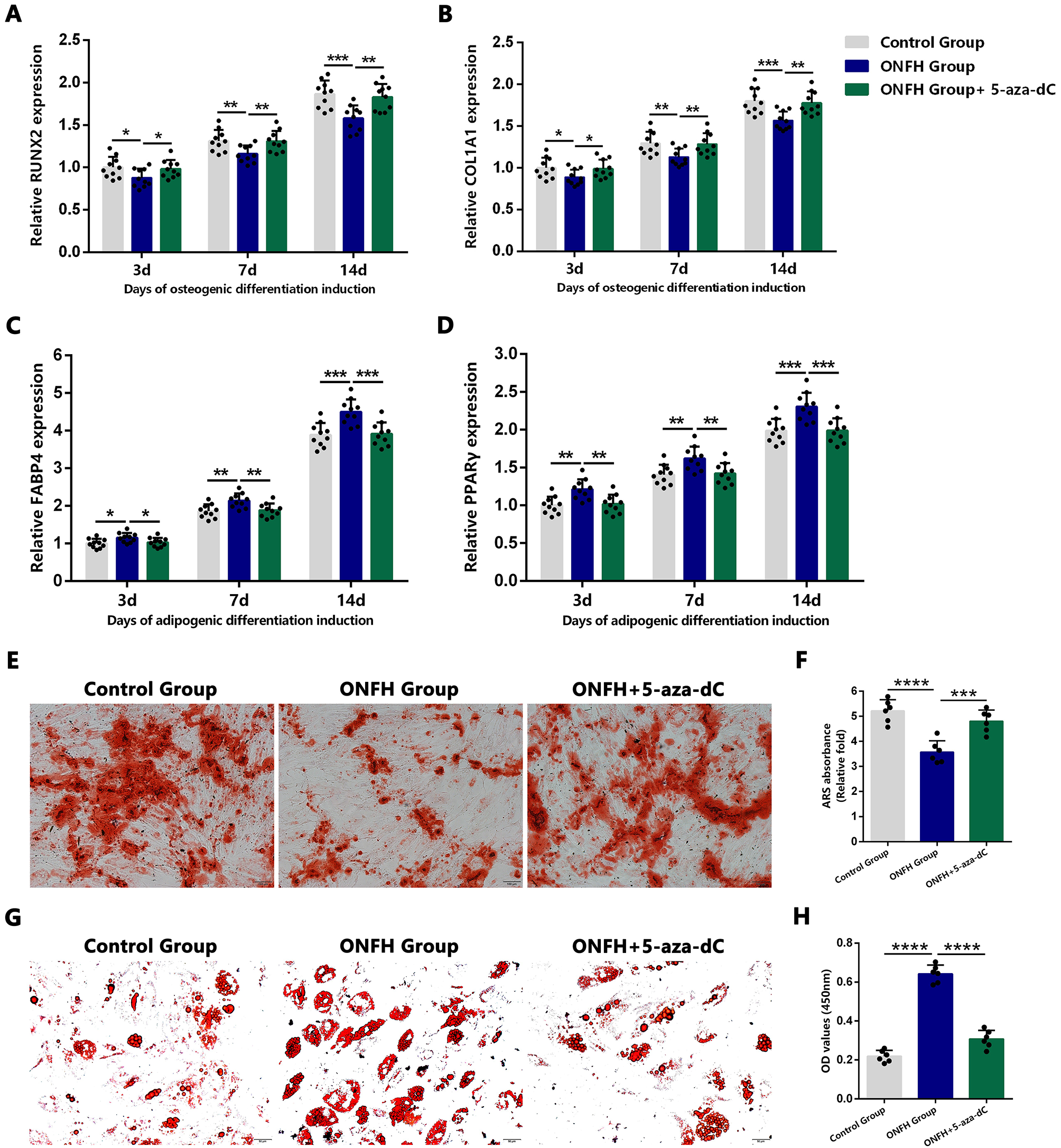

Supplement: S2 Fig — (A–B) qRT-PCR analysis revealed the relative expression of osteogenic marker genes, RUNX2 (A) and COL1A1 (B), in hBMSCs at days 3, 7, and 14 during osteogenic differentiation (n = 10). (C–D) qRT-PCR analysis showed the relative expression of adipogenic marker genes, FABP4 (C) and PPARγ (D), in hBMSCs at days 3, 7, and 14 during adipogenic differentiation (n = 10). (E–F) ARS staining (E) and quantification (F) were performed to measure the calcium deposits for matrix mineralization in hBMSCs after 14 days of osteogenic induction (n = 6). Scale bars: 100 μm. (G–H) ORO staining (G) and quantification (H) were used to evaluate intracellular lipid accumulation in hBMSCs after 21 days of adipogenic induction (n = 6). Scale bars: 50 μm. Statistical analysis was performed using one/two-way ANOVA with Bonferroni’s post-hoc test. Data are presented as mean ± SD, *P < 0.05, **P < 0.01, ***P < 0.001, ****P < 0.0001. (TIF) [file pone.0345372.s002.tif]

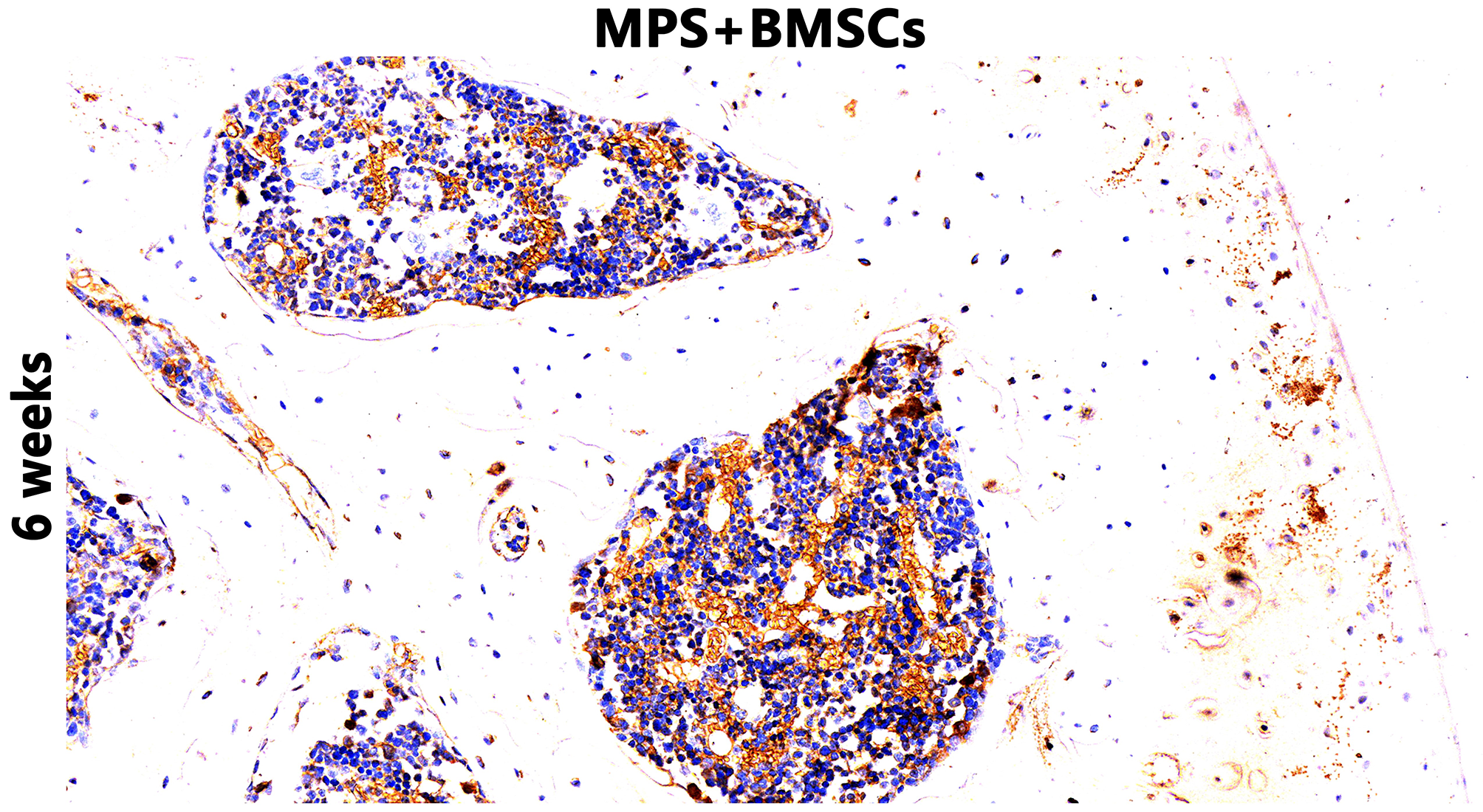

Supplement: S3 Fig — Scale bars: 50 μm. (TIF) [file pone.0345372.s003.tif]

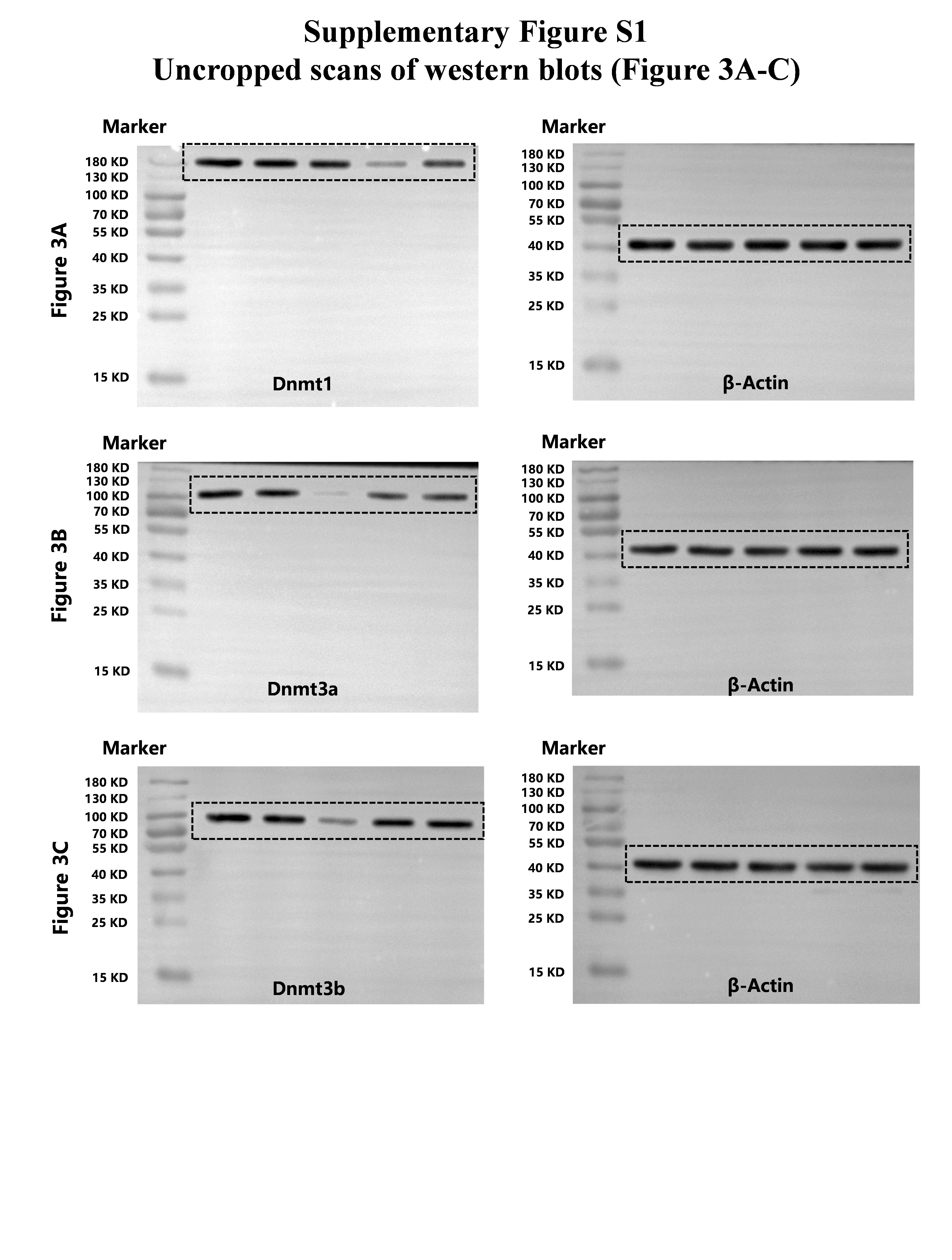

Supplement: S1 File — (ZIP) [file pone.0345372.s011.zip › S1_File/Additional file 5 (Supplementary Figure S1).jpg]

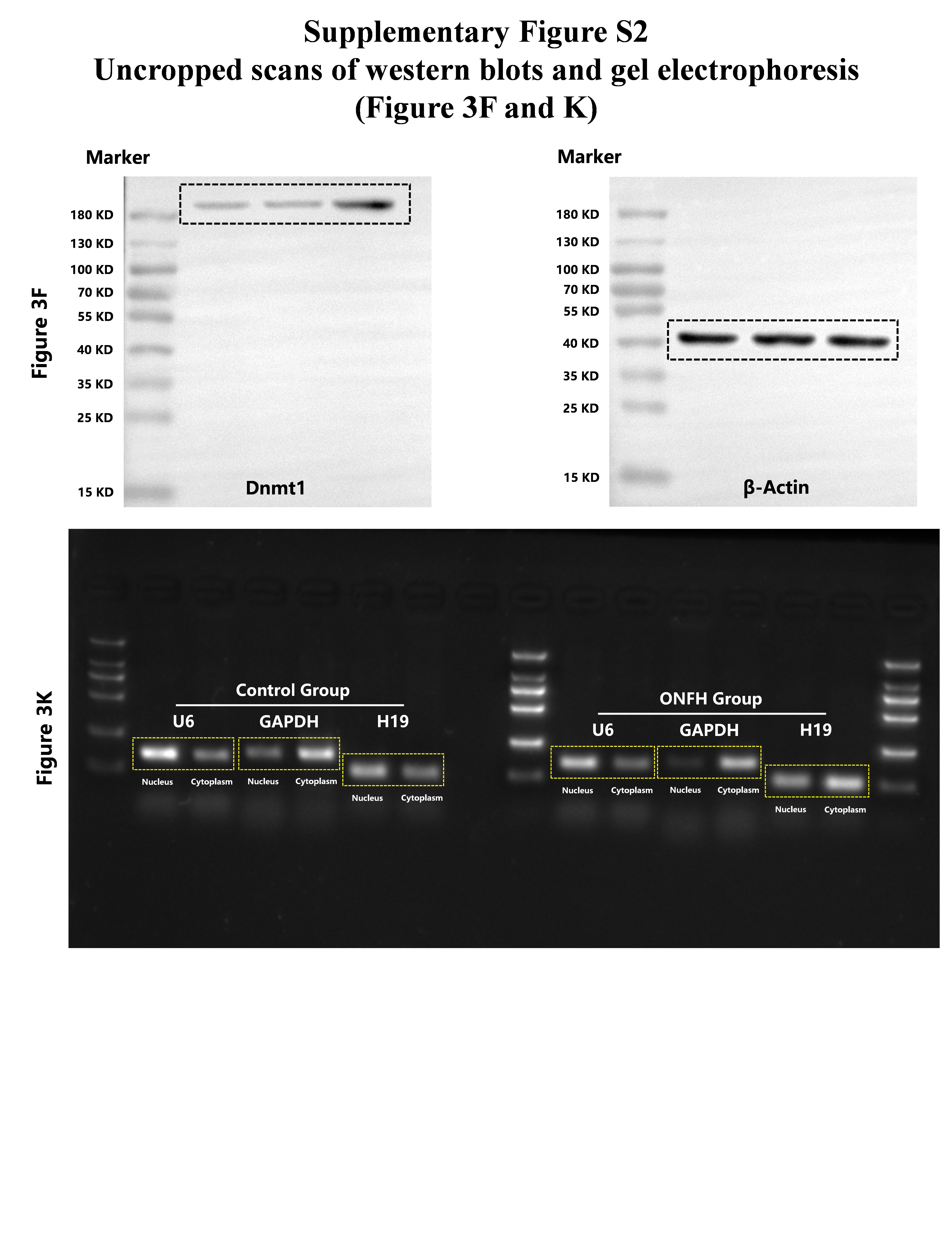

Supplement: S1 File — (ZIP) [file pone.0345372.s011.zip › S1_File/Additional file 5 (Supplementary Figure S2).jpg]

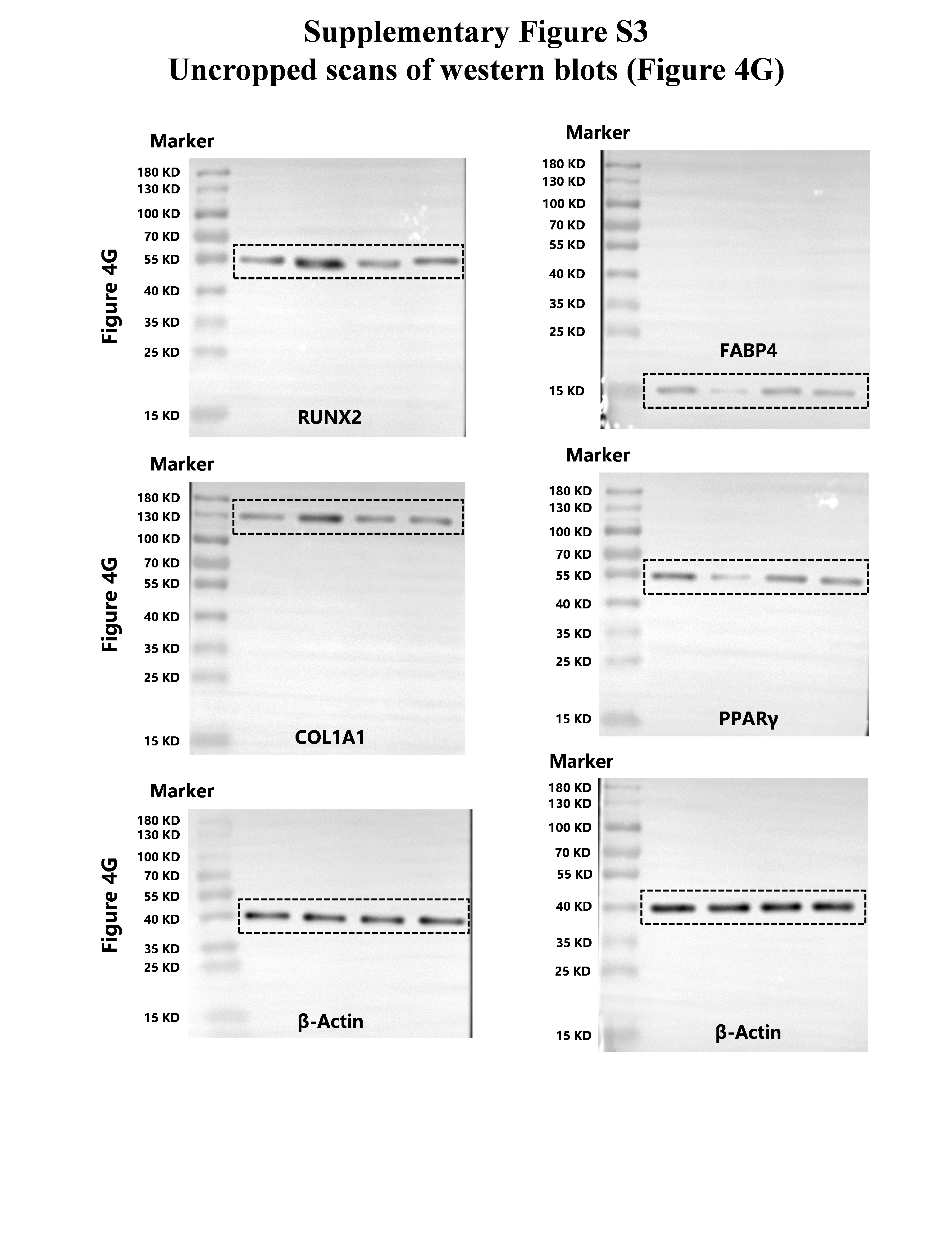

Supplement: S1 File — (ZIP) [file pone.0345372.s011.zip › S1_File/Additional file 5 (Supplementary Figure S3).jpg]

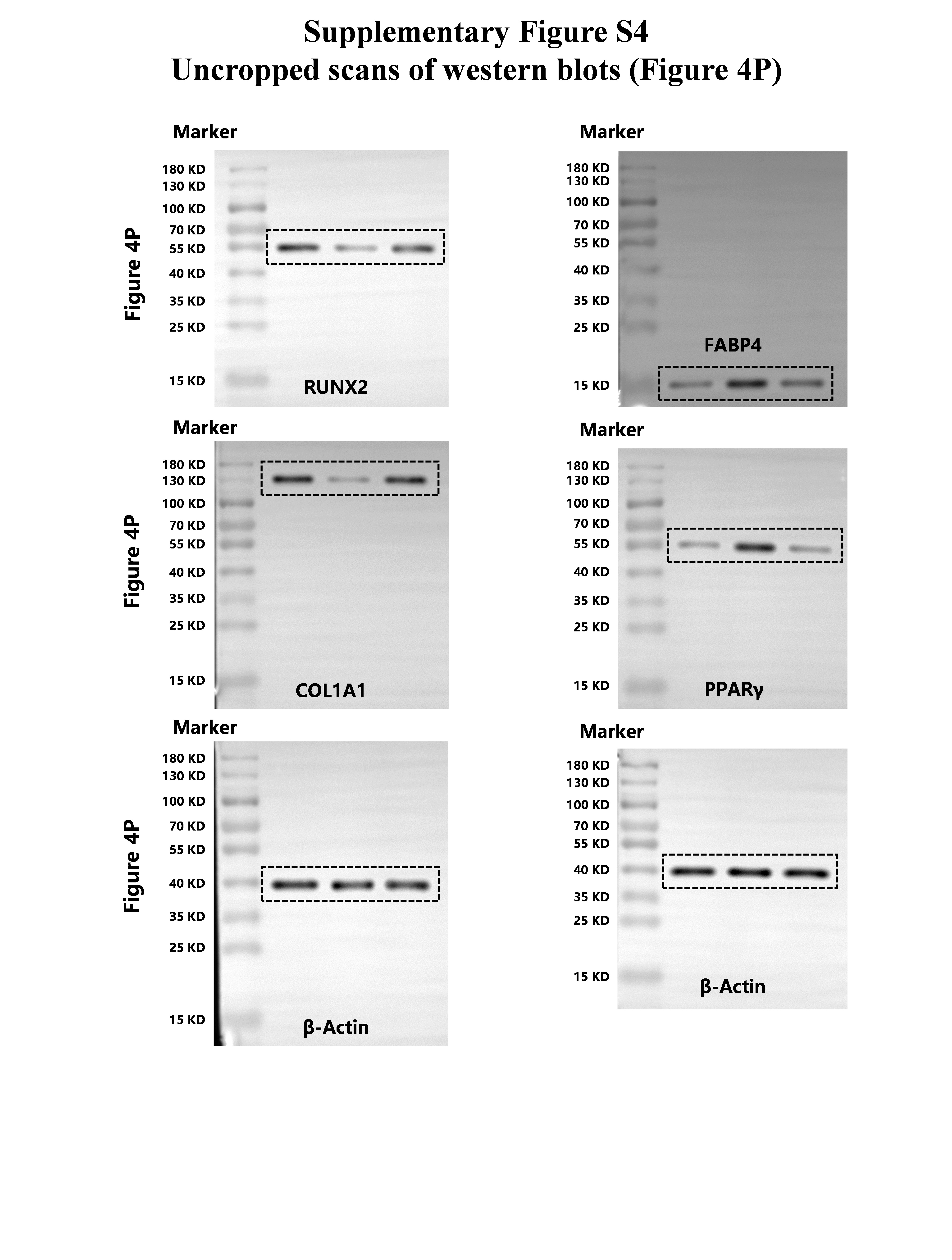

Supplement: S1 File — (ZIP) [file pone.0345372.s011.zip › S1_File/Additional file 5 (Supplementary Figure S4).jpg]

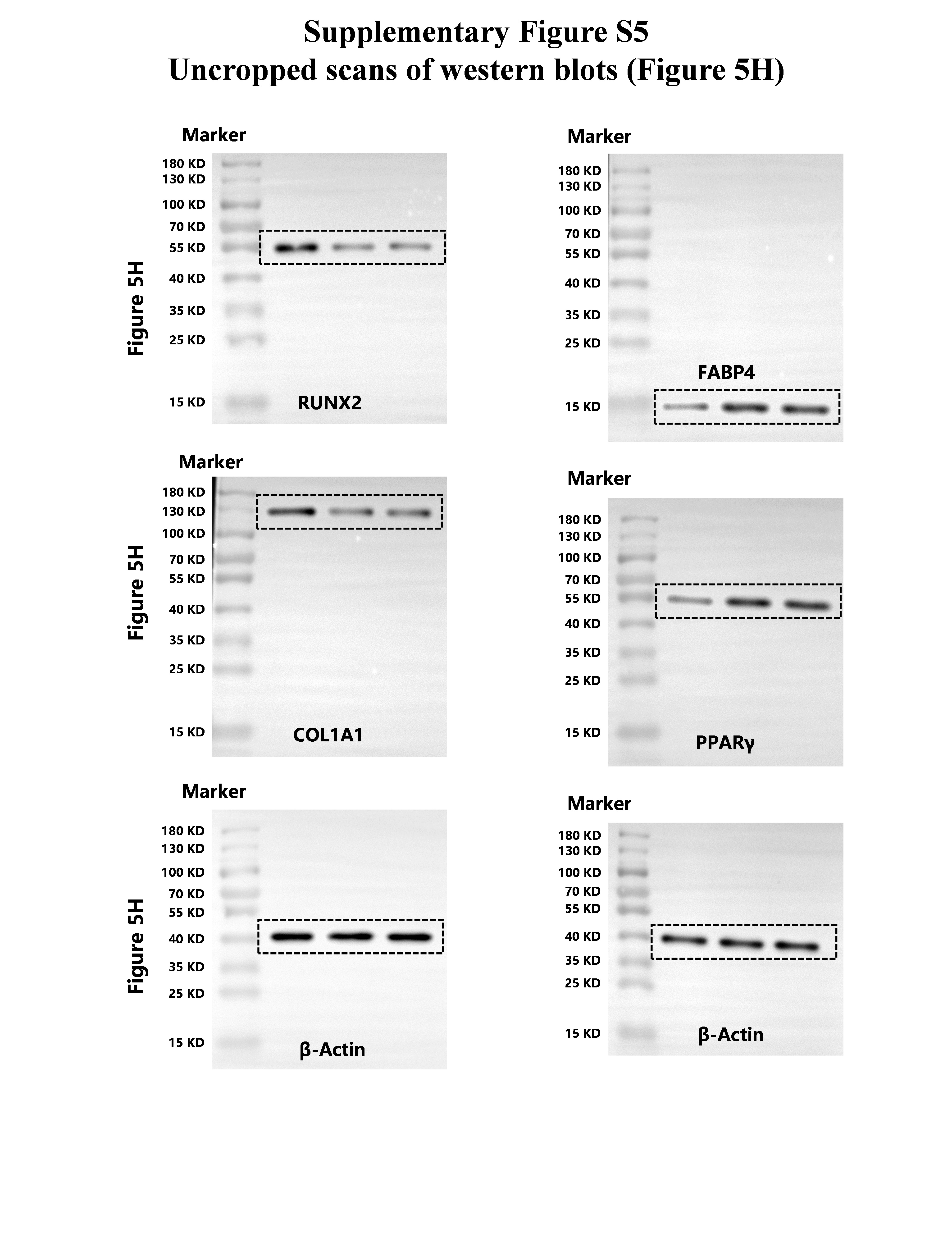

Supplement: S1 File — (ZIP) [file pone.0345372.s011.zip › S1_File/Additional file 5 (Supplementary Figure S5).jpg]

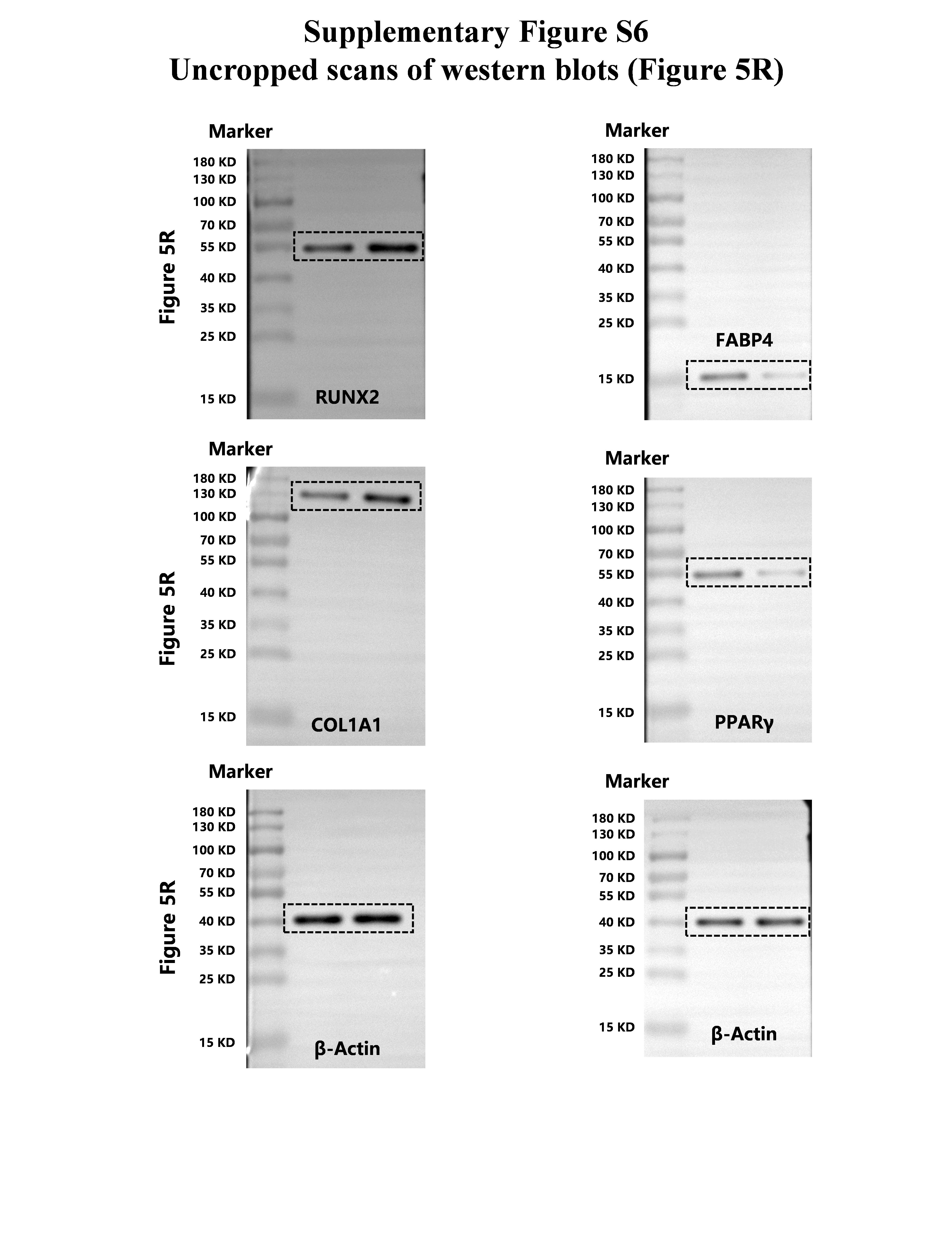

Supplement: S1 File — (ZIP) [file pone.0345372.s011.zip › S1_File/Additional file 5 (Supplementary Figure S6).jpg]

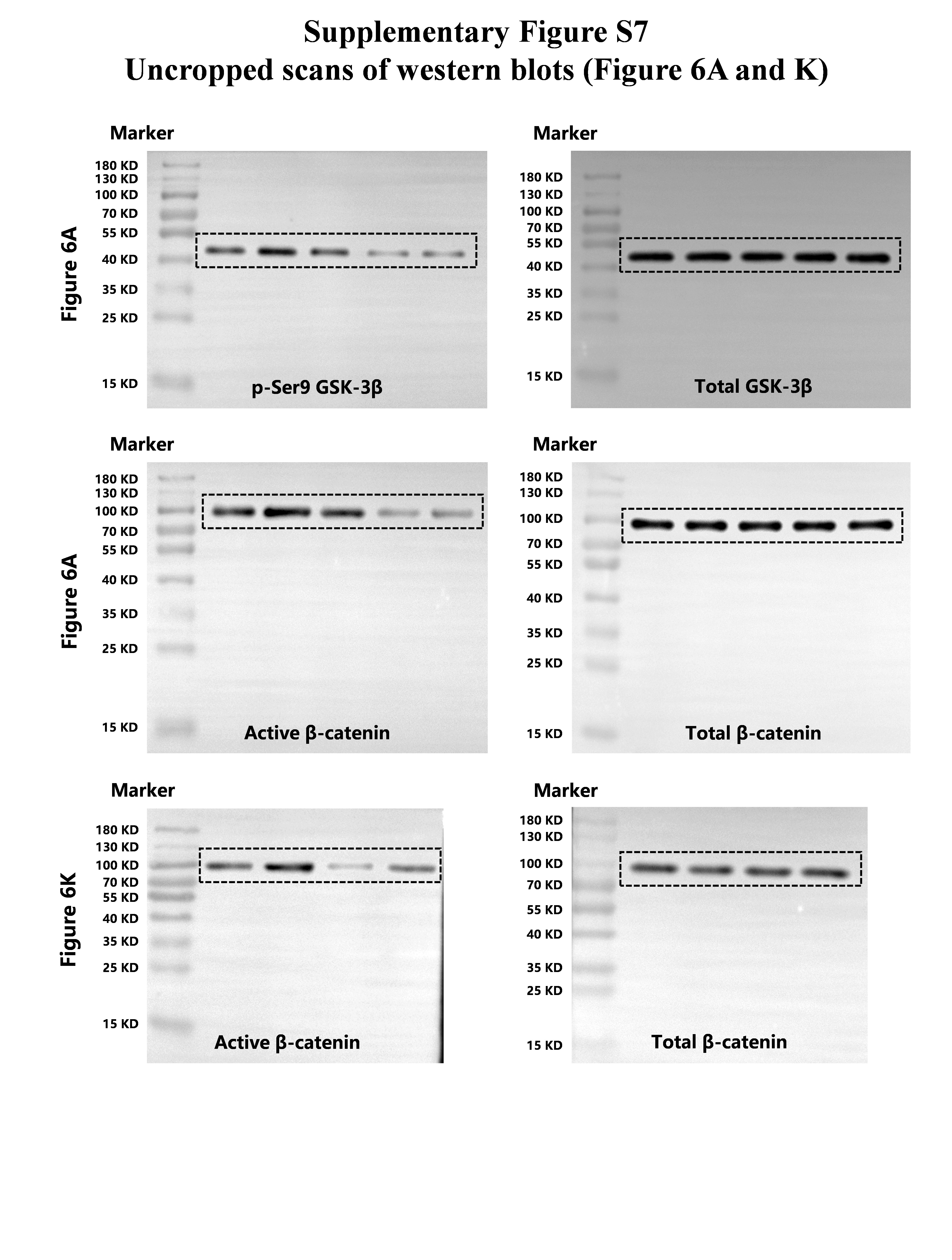

Supplement: S1 File — (ZIP) [file pone.0345372.s011.zip › S1_File/Additional file 5 (Supplementary Figure S7).jpg]

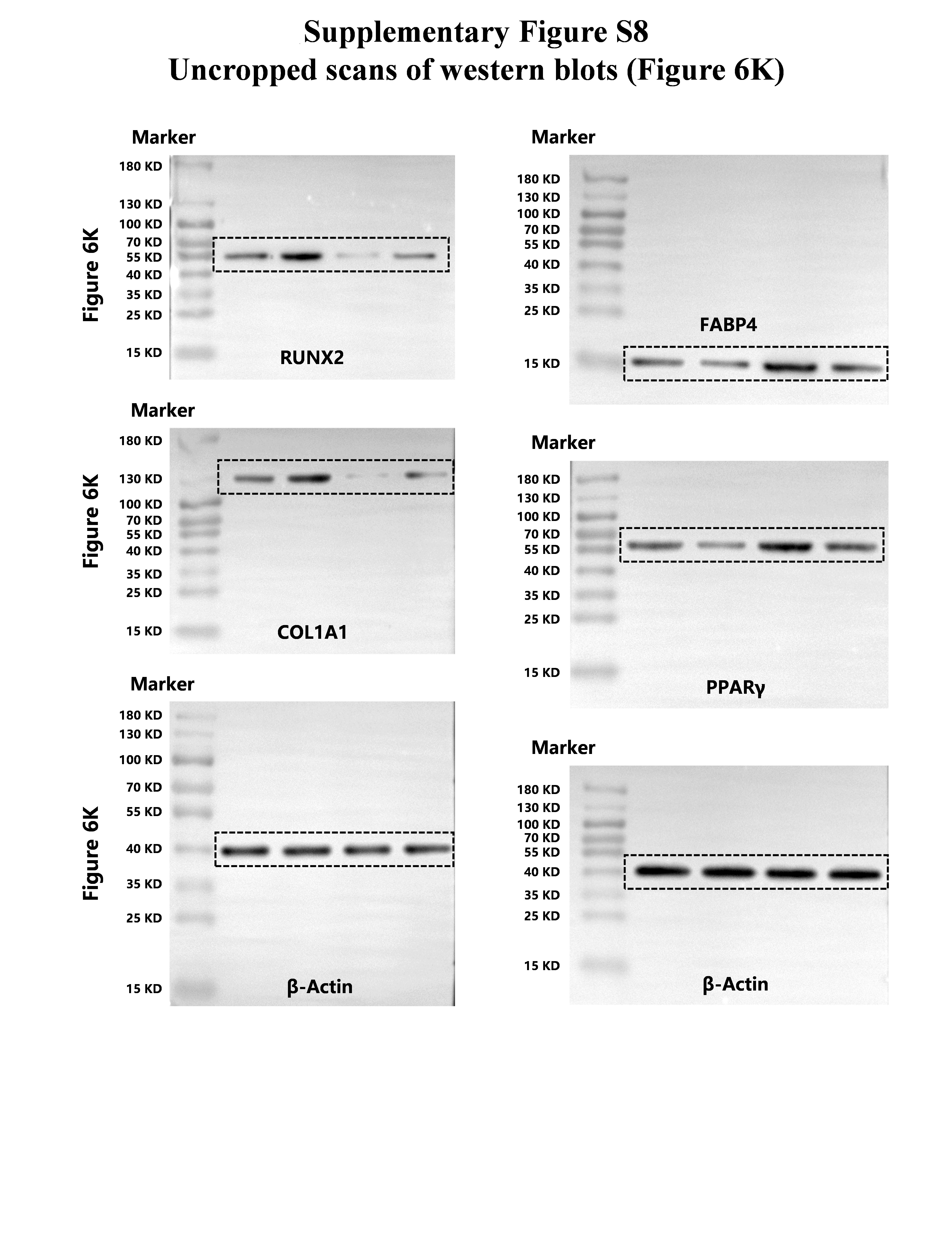

Supplement: S1 File — (ZIP) [file pone.0345372.s011.zip › S1_File/Additional file 5 (Supplementary Figure S8).jpg]

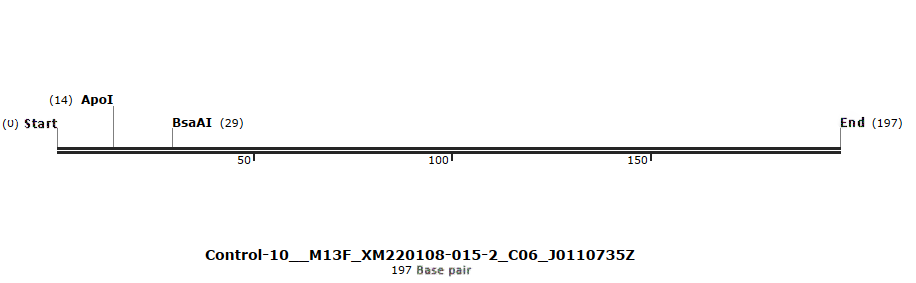

Supplement: S2 File — (ZIP) [file pone.0345372.s012.zip › Bisulfite sequencing/Control group/Control-10__M13F_XM220108-015-2_C06_J0110735Z.png]

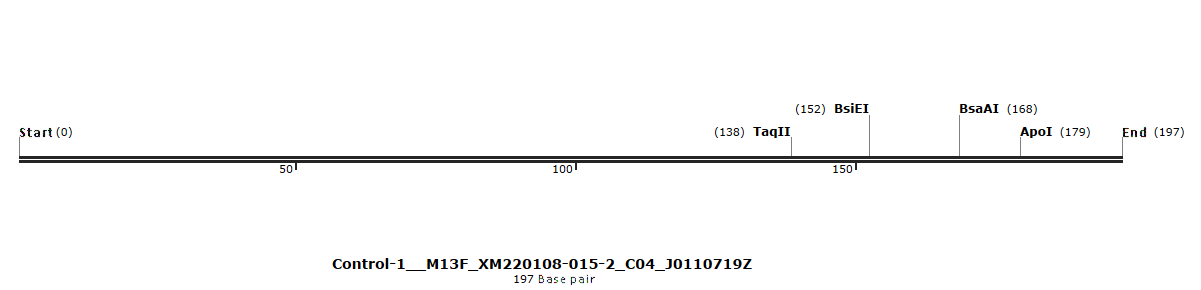

Supplement: S2 File — (ZIP) [file pone.0345372.s012.zip › Bisulfite sequencing/Control group/Control-1__M13F_XM220108-015-2_C04_J0110719Z.png]

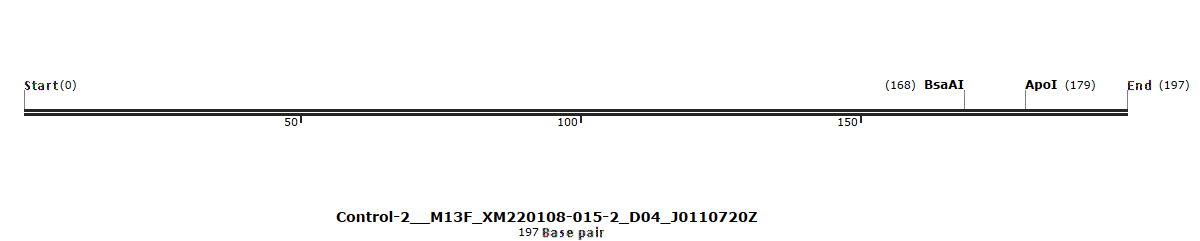

Supplement: S2 File — (ZIP) [file pone.0345372.s012.zip › Bisulfite sequencing/Control group/Control-2__M13F_XM220108-015-2_D04_J0110720Z.png]

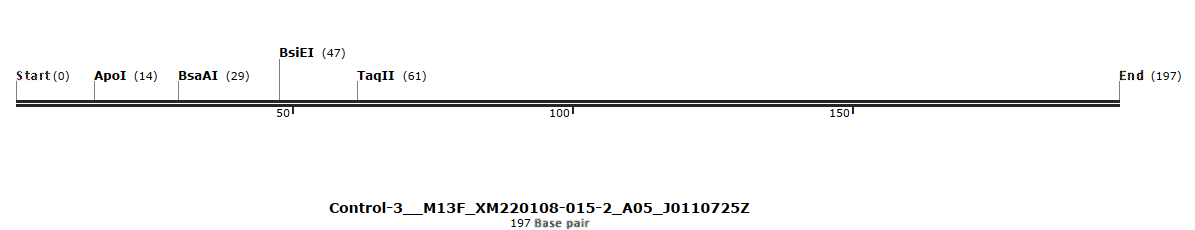

Supplement: S2 File — (ZIP) [file pone.0345372.s012.zip › Bisulfite sequencing/Control group/Control-3__M13F_XM220108-015-2_A05_J0110725Z.png]

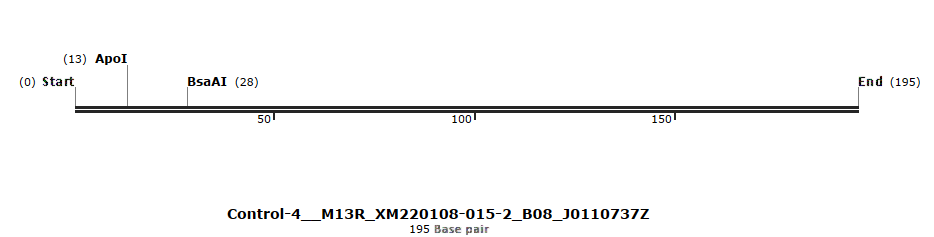

Supplement: S2 File — (ZIP) [file pone.0345372.s012.zip › Bisulfite sequencing/Control group/Control-4__M13R_XM220108-015-2_B08_J0110737Z.png]

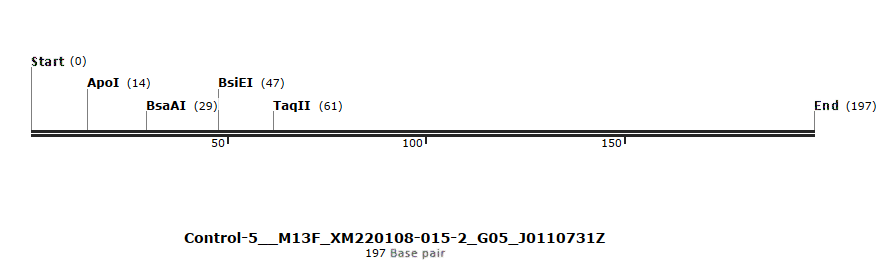

Supplement: S2 File — (ZIP) [file pone.0345372.s012.zip › Bisulfite sequencing/Control group/Control-5__M13F_XM220108-015-2_G05_J0110731Z.png]

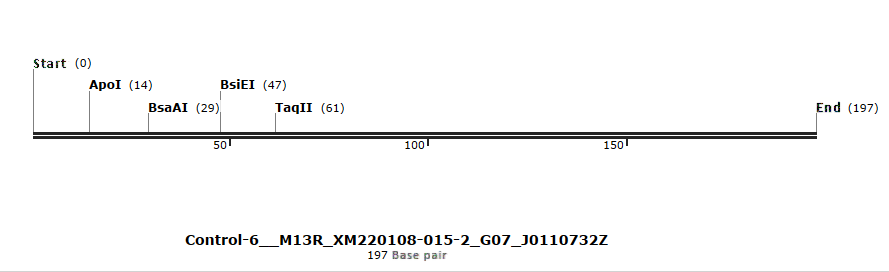

Supplement: S2 File — (ZIP) [file pone.0345372.s012.zip › Bisulfite sequencing/Control group/Control-6__M13R_XM220108-015-2_G07_J0110732Z.png]

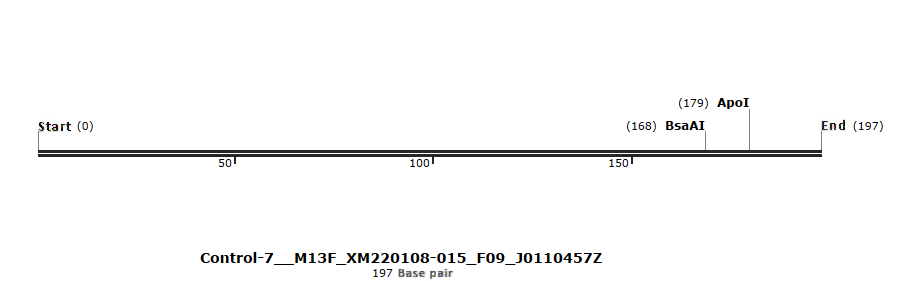

Supplement: S2 File — (ZIP) [file pone.0345372.s012.zip › Bisulfite sequencing/Control group/Control-7__M13F_XM220108-015_F09_J0110457Z.png]

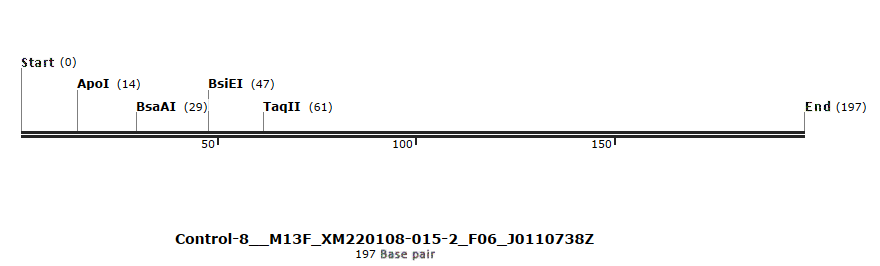

Supplement: S2 File — (ZIP) [file pone.0345372.s012.zip › Bisulfite sequencing/Control group/Control-8__M13F_XM220108-015-2_F06_J0110738Z.png]

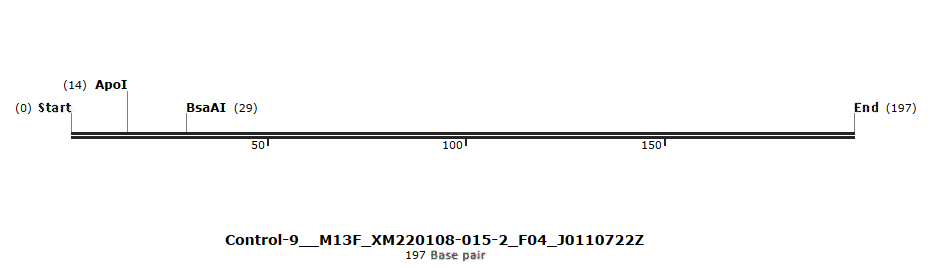

Supplement: S2 File — (ZIP) [file pone.0345372.s012.zip › Bisulfite sequencing/Control group/Control-9__M13F_XM220108-015-2_F04_J0110722Z.png]

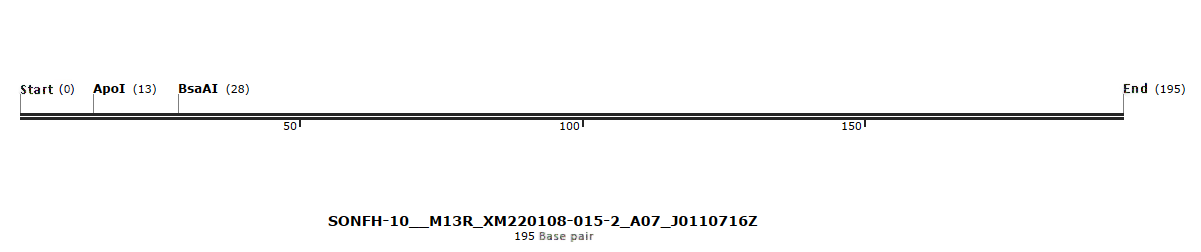

Supplement: S2 File — (ZIP) [file pone.0345372.s012.zip › Bisulfite sequencing/SONFH group/SONFH-10__M13R_XM220108-015-2_A07_J0110716Z.png]

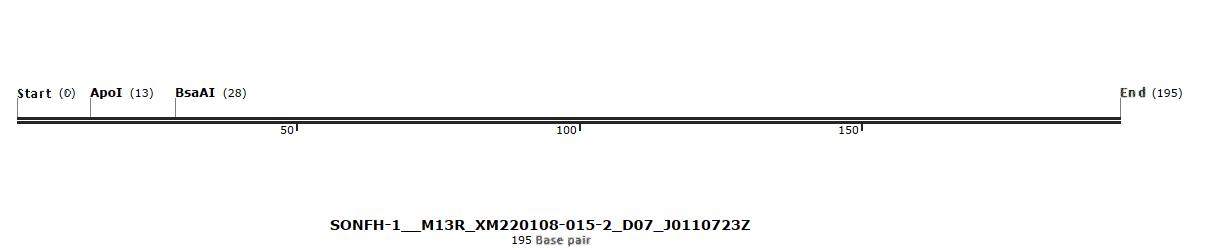

Supplement: S2 File — (ZIP) [file pone.0345372.s012.zip › Bisulfite sequencing/SONFH group/SONFH-1__M13R_XM220108-015-2_D07_J0110723Z.png]

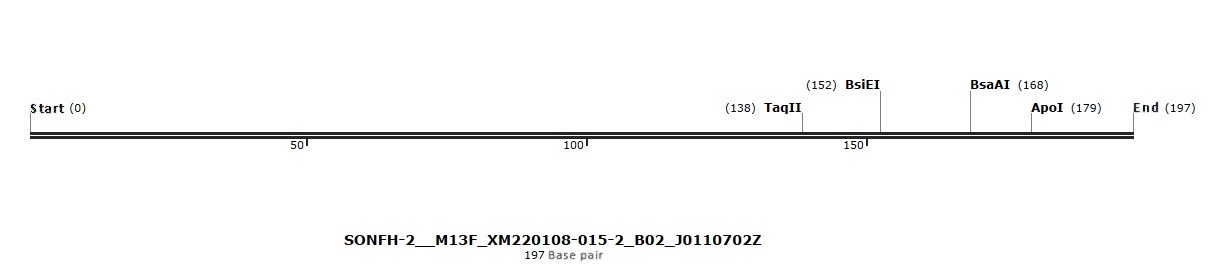

Supplement: S2 File — (ZIP) [file pone.0345372.s012.zip › Bisulfite sequencing/SONFH group/SONFH-2__M13F_XM220108-015-2_B02_J0110702Z.png]

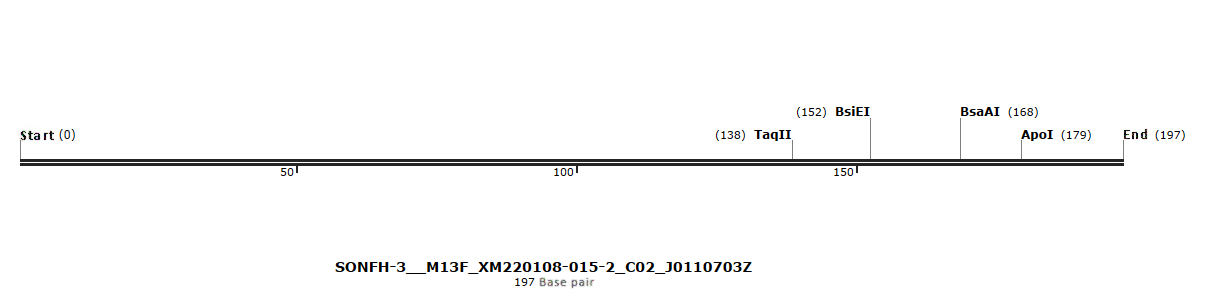

Supplement: S2 File — (ZIP) [file pone.0345372.s012.zip › Bisulfite sequencing/SONFH group/SONFH-3__M13F_XM220108-015-2_C02_J0110703Z.png]

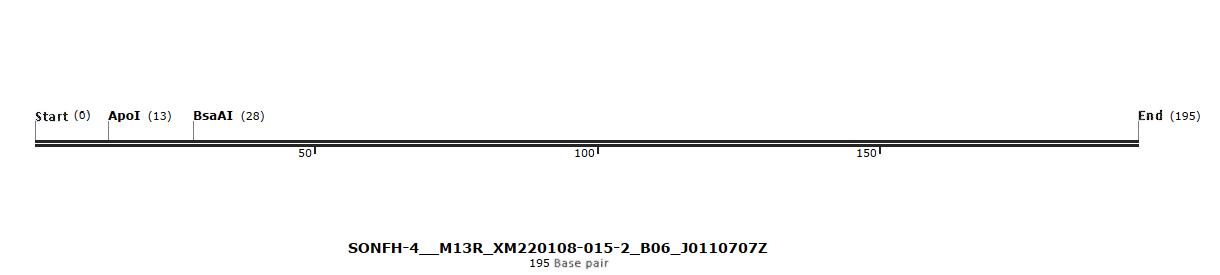

Supplement: S2 File — (ZIP) [file pone.0345372.s012.zip › Bisulfite sequencing/SONFH group/SONFH-4__M13R_XM220108-015-2_B06_J0110707Z.png]

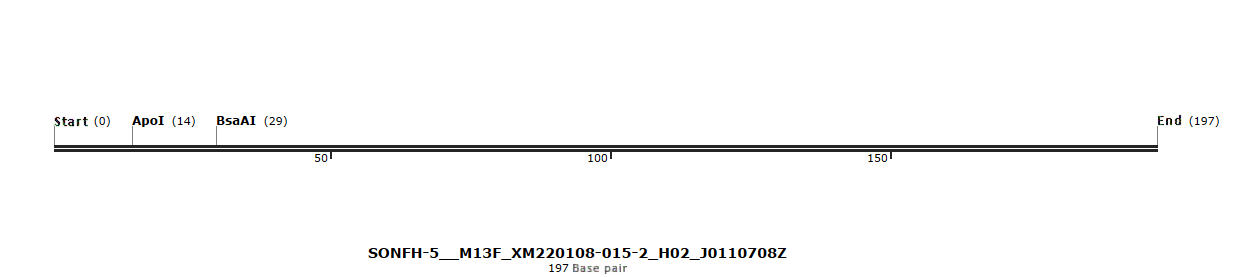

Supplement: S2 File — (ZIP) [file pone.0345372.s012.zip › Bisulfite sequencing/SONFH group/SONFH-5__M13F_XM220108-015-2_H02_J0110708Z.png]

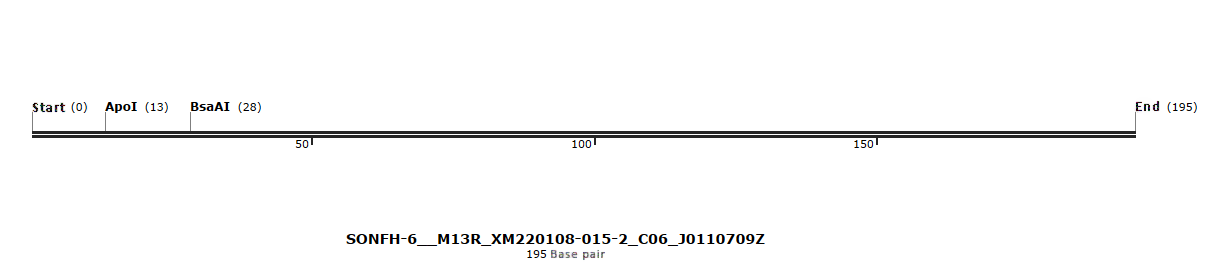

Supplement: S2 File — (ZIP) [file pone.0345372.s012.zip › Bisulfite sequencing/SONFH group/SONFH-6__M13R_XM220108-015-2_C06_J0110709Z.png]

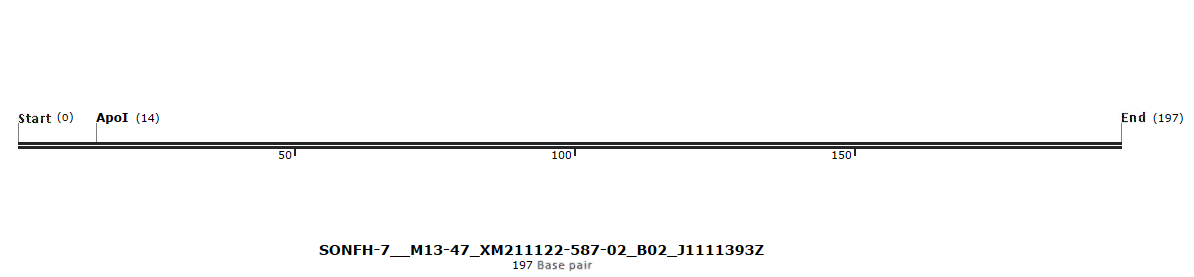

Supplement: S2 File — (ZIP) [file pone.0345372.s012.zip › Bisulfite sequencing/SONFH group/SONFH-7__M13-47_XM211122-587-02_B02_J1111393Z.png]

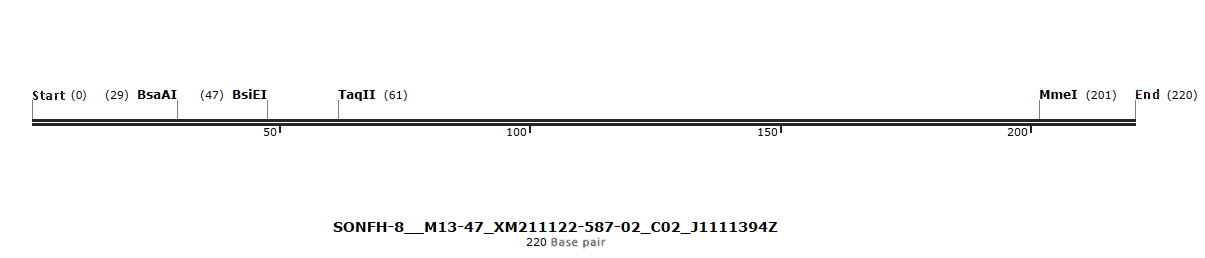

Supplement: S2 File — (ZIP) [file pone.0345372.s012.zip › Bisulfite sequencing/SONFH group/SONFH-8__M13-47_XM211122-587-02_C02_J1111394Z.png]

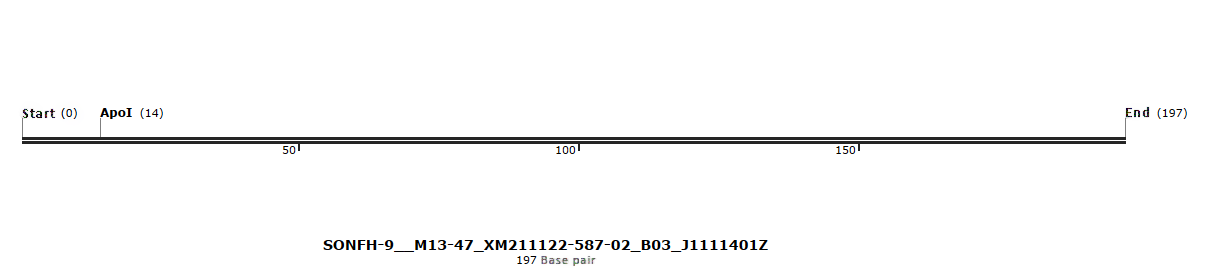

Supplement: S2 File — (ZIP) [file pone.0345372.s012.zip › Bisulfite sequencing/SONFH group/SONFH-9__M13-47_XM211122-587-02_B03_J1111401Z.png]

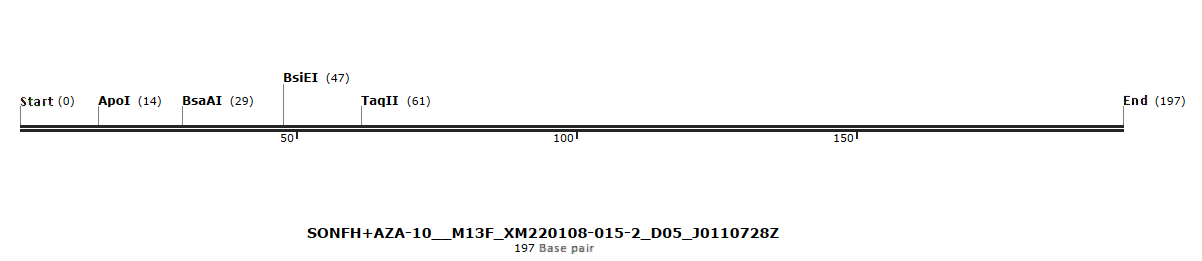

Supplement: S2 File — (ZIP) [file pone.0345372.s012.zip › Bisulfite sequencing/SONFH+5'AZA group/SONFH+AZA-10__M13F_XM220108-015-2_D05_J0110728Z.png]

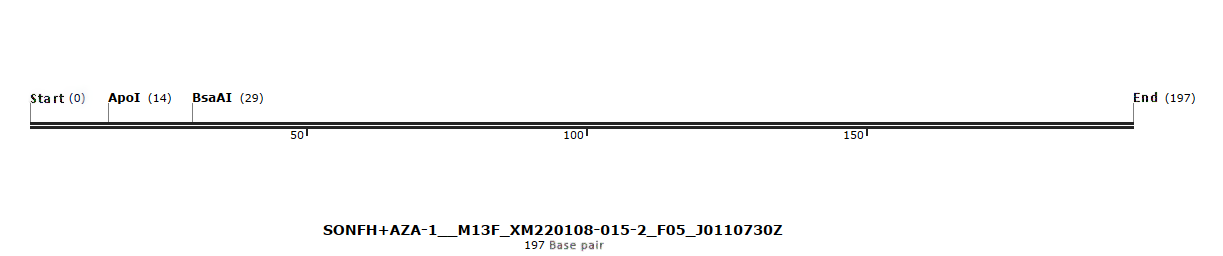

Supplement: S2 File — (ZIP) [file pone.0345372.s012.zip › Bisulfite sequencing/SONFH+5'AZA group/SONFH+AZA-1__M13F_XM220108-015-2_F05_J0110730Z.png]

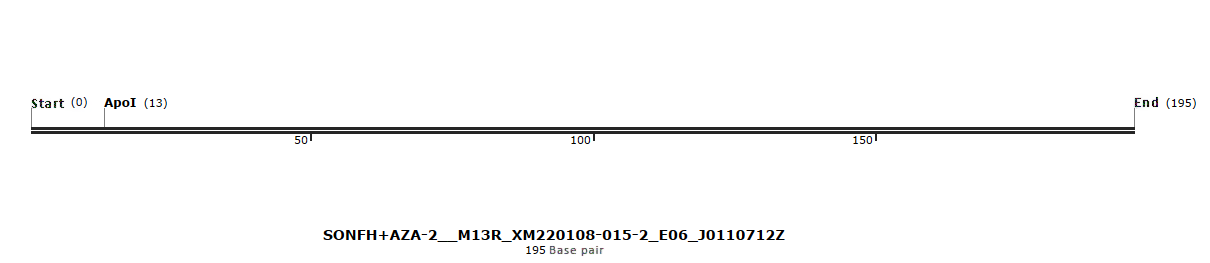

Supplement: S2 File — (ZIP) [file pone.0345372.s012.zip › Bisulfite sequencing/SONFH+5'AZA group/SONFH+AZA-2__M13R_XM220108-015-2_E06_J0110712Z.png]

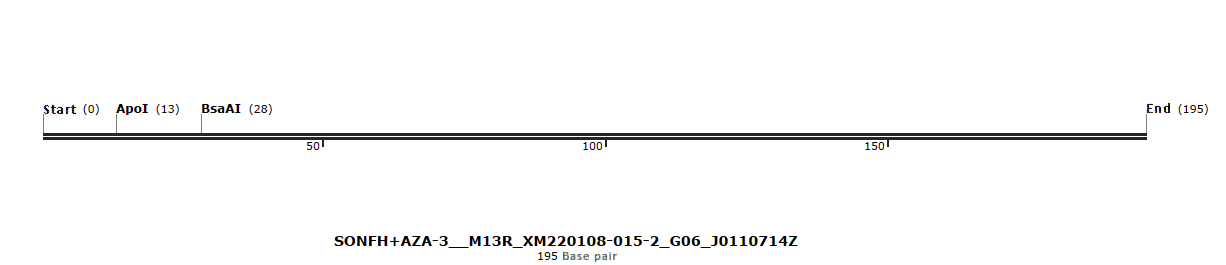

Supplement: S2 File — (ZIP) [file pone.0345372.s012.zip › Bisulfite sequencing/SONFH+5'AZA group/SONFH+AZA-3__M13R_XM220108-015-2_G06_J0110714Z.png]

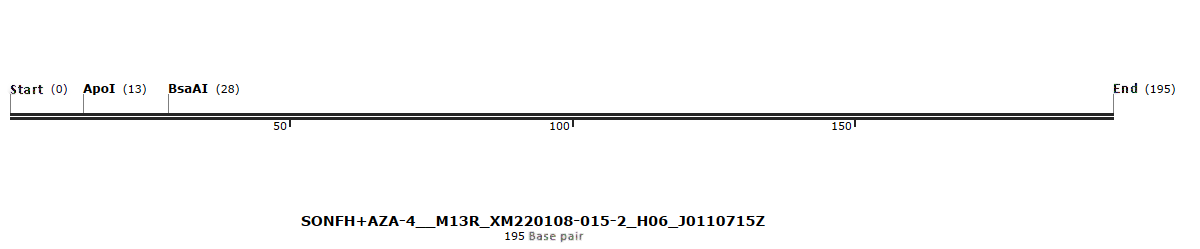

Supplement: S2 File — (ZIP) [file pone.0345372.s012.zip › Bisulfite sequencing/SONFH+5'AZA group/SONFH+AZA-4__M13R_XM220108-015-2_H06_J0110715Z.png]

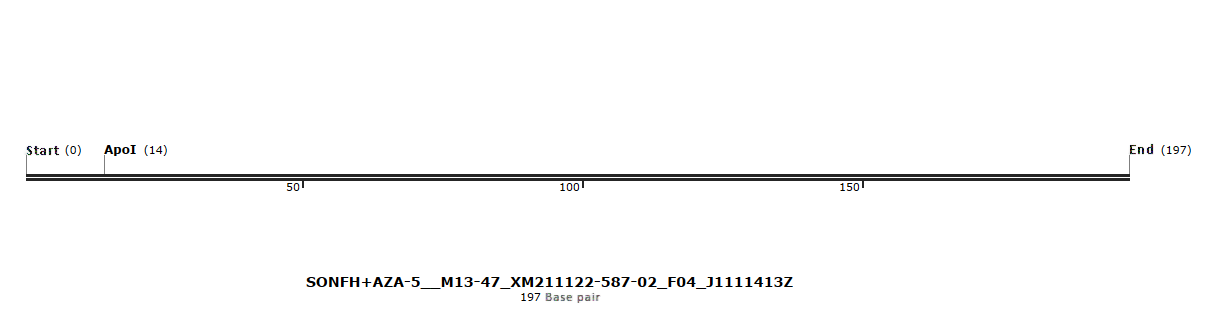

Supplement: S2 File — (ZIP) [file pone.0345372.s012.zip › Bisulfite sequencing/SONFH+5'AZA group/SONFH+AZA-5__M13-47_XM211122-587-02_F04_J1111413Z.png]

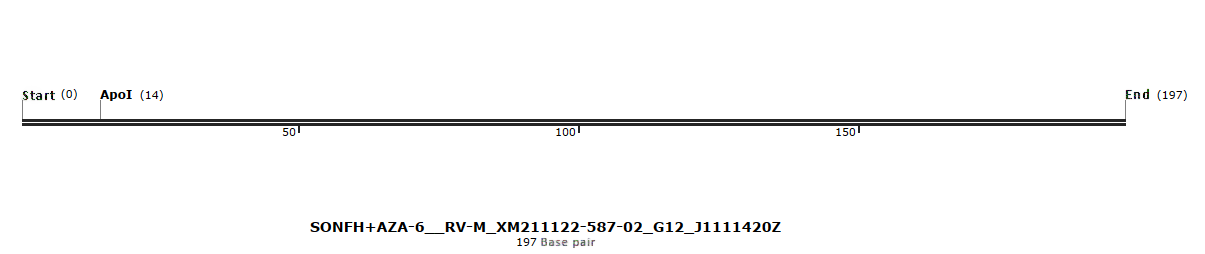

Supplement: S2 File — (ZIP) [file pone.0345372.s012.zip › Bisulfite sequencing/SONFH+5'AZA group/SONFH+AZA-6__RV-M_XM211122-587-02_G12_J1111420Z.png]

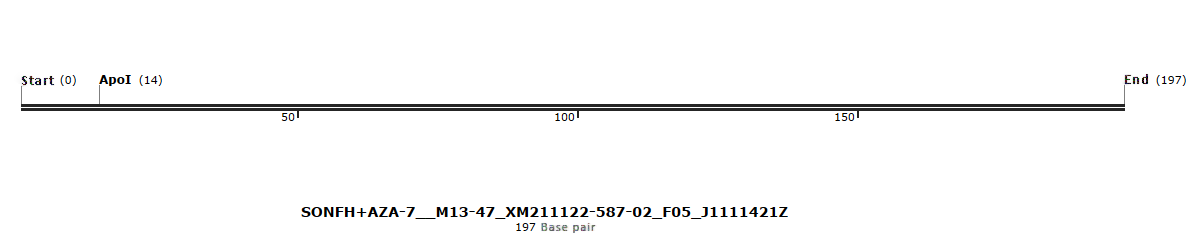

Supplement: S2 File — (ZIP) [file pone.0345372.s012.zip › Bisulfite sequencing/SONFH+5'AZA group/SONFH+AZA-7__M13-47_XM211122-587-02_F05_J1111421Z.png]

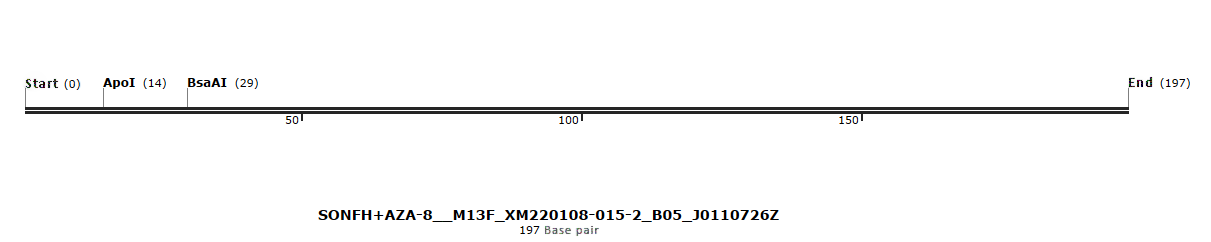

Supplement: S2 File — (ZIP) [file pone.0345372.s012.zip › Bisulfite sequencing/SONFH+5'AZA group/SONFH+AZA-8__M13F_XM220108-015-2_B05_J0110726Z.png]

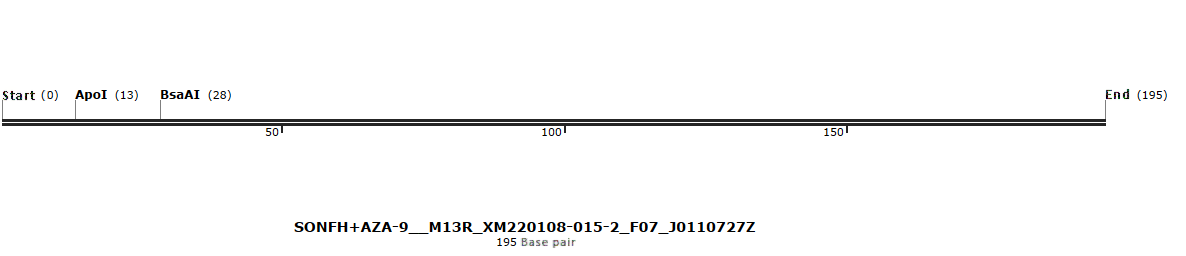

Supplement: S2 File — (ZIP) [file pone.0345372.s012.zip › Bisulfite sequencing/SONFH+5'AZA group/SONFH+AZA-9__M13R_XM220108-015-2_F07_J0110727Z.png]
