# Supplementary material for: Glucocorticoid-Induced alterations in DNA methylation in the H19 promoter of Bone Marrow-Derived Mesenchymal Stem Cells are associated with the pathogenesis of osteonecrosis
Source: PLoS One. 2026 Mar 27;21(3):e0345372. doi: 10.1371/journal.pone.0345372 (PMC13028513; doi:10.1371/journal.pone.0345372)
Supplement: S1 Table — (DOCX) [file pone.0345372.s004.docx]

| **Table S1** Detailed demographic and clinical characteristics of each patient from the control group and the ONFH group. | | | | | | |
| --- | --- | --- | --- | --- | --- | --- |
| **No.** | **Gender/Age ranges** | **diagnosis** | **Hight (cm)** | **Weigh (kg)** | **BMI** | **B.G. (mmol/L)** |
| 1 | F/56-60 | FNF | 163 | 70 | 26.35 | 6.2 |
| 2 | F/56-60 | FNF | 155 | 55 | 22.89 | 3.4 |
| 3 | F/46-50 | FNF | 163 | 57 | 21.45 | 4.5 |
| 4 | M/51-55 | FNF | 167 | 60 | 21.51 | 5.1 |
| 5 | F/56-60 | FNF | 168 | 60 | 21.26 | 6 |
| 6 | M/51-55 | FNF | 178 | 73 | 23.04 | 5.8 |
| 7 | F/41-45 | FNF | 160 | 55 | 21.48 | 5 |
| 8 | M/51-55 | FNF | 175 | 65 | 21.22 | 5.2 |
| 9 | M/56-60 | FNF | 175 | 70 | 22.86 | 4.7 |
| 10 | M/41-45 | FNF | 186 | 75 | 21.68 | 4.8 |
| 11 | F/41-45 | GC-induced ONFH | 163 | 60 | 22.58 | 4.6 |
| 12 | M/56-60 | GC-induced ONFH | 168 | 75 | 26.57 | 5.1 |
| 13 | F/56-60 | GC-induced ONFH | 160 | 57 | 22.27 | 4.3 |
| 14 | F/56-60 | GC-induced ONFH | 165 | 61 | 22.41 | 5.1 |
| 15 | M/56-60 | GC-induced ONFH | 169 | 70 | 24.51 | 5.4 |
| 16 | M/51-55 | GC-induced ONFH | 170 | 70 | 24.22 | 4.7 |
| 17 | F/46-50 | GC-induced ONFH | 158 | 56 | 22.43 | 4.1 |
| 18 | F/41-45 | GC-induced ONFH | 167 | 73 | 26.18 | 5.2 |
| 19 | M/51-55 | GC-induced ONFH | 173 | 76 | 25.39 | 4.8 |
| 20 | M/51-55 | GC-induced ONFH | 182 | 78 | 23.55 | 4.9 |
| FNF: Femoral Neck Fracture; ONFH: Osteonecrosis of femoral head; B.G., Blood Glucose | | | | | | |
