# Supplementary material for: Glucocorticoid-Induced alterations in DNA methylation in the H19 promoter of Bone Marrow-Derived Mesenchymal Stem Cells are associated with the pathogenesis of osteonecrosis
Source: PLoS One. 2026 Mar 27;21(3):e0345372. doi: 10.1371/journal.pone.0345372 (PMC13028513; doi:10.1371/journal.pone.0345372)
Supplement: S2 Table — (DOCX) [file pone.0345372.s005.docx]

| **Table S2** The primer sequences used for qRT-PCR quantifications. | | | |
| --- | --- | --- | --- |
| **Gene** | **Origin** | **Forward (5’→3’)** | **Reverse (5’→3’)** |
| H19 | human | ACCAGCCACCACATCATC | TCAGAAACAAAGAGACAGAAGGA |
| Dnmt1 | human | AGAACGGTGCTCATGCTTACA | CTCTACGGGCTTCACTTCTTG |
| Dnmt3a | human | CCGATGCTGGGGACAAGAAT | CCCGTCATCCACCAAGACAC |
| Dnmt3b | human | TAACAACGGCAAAGACCGAGGG | TCCTGCCACAAGACAAACAGCC |
| RUNX2 | human | CCGCCTCAGTGATTTAGGGC | GGGTCTGTAATCTGACTCTGTCC |
| COL1A1 | human | GTGCGATGACGTGATCTGTGA | CGGTGGTTTCTTGGTCGGT |
| FABP4 | human | ACGAGAGGATGATAAACTGGTGG | GCGAACTTCAGTCCAGGTCAAC |
| PPARγ | human | ACCAAAGTGCAATCAAAGTGGA | ATGAGGGAGTTGGAAGGCTCT |
| β-actin | human | TGGCACCCAGCACAATGAA | CTAAGTCATAGTCCGCCTAGAAGCA |
| H19 | rats | TGACCTCCCAACAGAATGGC | AAAGCTCTTTCCACTCCGGG |
| β-actin | rats | CTGTGTGGATTGGTGGCTCT | CAGCTCAGTAACAGTCCGCC |
