# Supplementary material for: Glucocorticoid-Induced alterations in DNA methylation in the H19 promoter of Bone Marrow-Derived Mesenchymal Stem Cells are associated with the pathogenesis of osteonecrosis
Source: PLoS One. 2026 Mar 27;21(3):e0345372. doi: 10.1371/journal.pone.0345372 (PMC13028513; doi:10.1371/journal.pone.0345372)
Supplement: S3 Table — (DOCX) [file pone.0345372.s006.docx]

| **Table S3** siRNA sequences used for gene silencing. | | |  |
| --- | --- | --- | --- |
| **Genes** | **Species** | **Sense** | **Antisense** |
| si-Dnmt1-1 | human | GAAGAGACGUAGAGUUACATT | UGUAACUCUACGUCUCUUCTT |
| si-Dnmt1-2 | human | GGAACUUUGUCUCCUUCAATT | UUGAAGGAGACAAAGUUCCTT |
| si-Dnmt1-3 | human | CAAUGAGACUGACAUCAAATT | UUUGAUGUCAGUCUCAUUGTT |
| si-Dnmt3a-1 | human | CCACCAAAGCAGGCGAUGATT | UCAUCGCCUGCUUUGGUGGTT |
| si-Dnmt3a-2 | human | CCACGACAGCGAUGAGAGUTT | ACUCUCAUCGCUGUCGUGGTT |
| si-Dnmt3a-3 | human | GCCUGGAGCCACCAGAAGATT | UCUUCUGGUGGCUCCAGGCTT |
| si-Dnmt3b-1 | human | GGACUACUUUGCAUGUGAATT | UUCACAUGCAAAGUAGUCCTT |
| si-Dnmt3b-2 | human | GCAACGAUCUCUCAAAUGUTT | ACAUUUGAGAGAUCGUUGCTT |
| si-Dnmt3b-3 | human | CCAAGCGCCUCAAGACAAATT | UUUGUCUUGAGGCGCUUGGTT |
| si-H19-1 | human | GGCCUUCCUGAACACCUUATT | UAAGGUGUUCAGGAAGGCCTT |
| si-H19-2 | human | GACGUGACAAGCAGGACAUTT | AUGUCCUGCUUGUCACGUCTT |
