# Supplementary material for: Glucocorticoid-Induced alterations in DNA methylation in the H19 promoter of Bone Marrow-Derived Mesenchymal Stem Cells are associated with the pathogenesis of osteonecrosis
Source: PLoS One. 2026 Mar 27;21(3):e0345372. doi: 10.1371/journal.pone.0345372 (PMC13028513; doi:10.1371/journal.pone.0345372)
Supplement: S4 Table — (DOCX) [file pone.0345372.s007.docx]

| **Table S4** shRNA sequences used for gene silencing. | | |  |
| --- | --- | --- | --- |
| **Genes** | **Species** | **Sense** | **Antisense** |
| sh-Dnmt1-1 | rats | GATGGCGTCATAACCAATAAA | TTTATTGGTTATGACGCCATC |
| sh-Dnmt1-2 | rats | CCAGAGTATGCACCAATATTT | AAATATTGGTGCATACTCTGG |
| sh-Dnmt1-3 | rats | ATTCCTGCAAACAGAAATAAA | TTTATTTCTGTTTGCAGGAAT |
